# Impaired Neurovascular Coupling and Increased Functional Connectivity in the Frontal Cortex Predict Age‐Related Cognitive Dysfunction

**DOI:** 10.1002/advs.202303516

**Published:** 2023-12-28

**Authors:** Peter Mukli, Camila B. Pinto, Cameron D. Owens, Tamas Csipo, Agnes Lipecz, Zsofia Szarvas, Anna Peterfi, Ana Clara da Costa Pinaffi Langley, Jordan Hoffmeister, Frigyes Samuel Racz, Jonathan W. Perry, Stefano Tarantini, Ádám Nyúl‐Tóth, Farzaneh A. Sorond, Yuan Yang, Judith A. James, Angelia C. Kirkpatrick, Calin I. Prodan, Peter Toth, Juliette Galindo, Andrew W. Gardner, William E. Sonntag, Anna Csiszar, Zoltan Ungvari, Andriy Yabluchanskiy

**Affiliations:** ^1^ Oklahoma Center for Geroscience and Healthy Brain Aging University of Oklahoma Health Sciences Center 1122 NE 13th Street Oklahoma City OK 73117 USA; ^2^ Vascular Cognitive Impairment and Neurodegeneration Program Department of Neurosurgery University of Oklahoma Health Sciences Center Oklahoma City OK 73117 USA; ^3^ Department of Physiology Faculty of Medicine Semmelweis University Budapest H‐1094 Hungary; ^4^ International Training Program in Geroscience Doctoral School of Basic and Translational Medicine/Department of Public Health Semmelweis University Budapest H‐1085 Hungary; ^5^ Department of Cardiology Division of Clinical Physiology Faculty of Medicine University of Debrecen Debrecen H‐4032 Hungary; ^6^ Department of Neurology Dell Medical School University of Texas at Austin Austin TX 78712 USA; ^7^ Department of Health Promotion Sciences College of Public Health University of Oklahoma Health Sciences Center Oklahoma City OK 73104 USA; ^8^ Peggy and Charles Stephenson Cancer Center University of Oklahoma Health Sciences Center Oklahoma City OK 73104 USA; ^9^ Department of Neurology Division of Stroke and Neurocritical Care Northwestern University Feinberg School of Medicine 303 E. Chicago Ave. Chicago IL 60611 USA; ^10^ Stephenson School of Biomedical Engineering The University of Oklahoma Tulsa OK 73019 USA; ^11^ Department of Physical Therapy and Human Movement Sciences Northwestern University Feinberg School of Medicine Chicago IL 60611 USA; ^12^ Department of Rehabilitation Sciences University of Oklahoma Health Science Center Oklahoma City OK 73117 USA; ^13^ Arthritis & Clinical Immunology Research Program Oklahoma Medical Research Foundation 825 NE 13^th^ Street Oklahoma City OK 73104 USA; ^14^ Department of Internal Medicine University of Oklahoma Health Sciences Center Oklahoma City OK 73117 USA; ^15^ Department of Pathology University of Oklahoma Health Sciences Center Oklahoma City OK 73104 USA; ^16^ Veterans Affairs Medical Center Oklahoma City OK 73104 USA; ^17^ Department of Neurology University of Oklahoma Health Sciences Center Oklahoma City OK 73104 USA; ^18^ Department of Neurosurgery Medical School University of Pecs Pecs H‐7623 Hungary; ^19^ Institute for Translational Medicine Medical School University of Pecs Pecs H‐7624 Hungary; ^20^ ELKH‐PTE Clinical Neuroscience MR Research Group Pecs H‐7624 Hungary; ^21^ Department of Physical Medicine and Rehabilitation Penn State College of Medicine 700 HMC Crescent Road Hershey PA 17033 USA

**Keywords:** aging, cognitive decline, functional connectivity, functional near‐infrared spectroscopy, neurovascular coupling

## Abstract

Impaired cerebrovascular function contributes to the genesis of age‐related cognitive decline. In this study, the hypothesis is tested that impairments in neurovascular coupling (NVC) responses and brain network function predict cognitive dysfunction in older adults. Cerebromicrovascular and working memory function of healthy young (*n* = 21, 33.2±7.0 years) and aged (*n* = 30, 75.9±6.9 years) participants are assessed. To determine NVC responses and functional connectivity (FC) during a working memory (n‐back) paradigm, oxy‐ and deoxyhemoglobin concentration changes from the frontal cortex using functional near‐infrared spectroscopy are recorded. NVC responses are significantly impaired during the 2‐back task in aged participants, while the frontal networks are characterized by higher local and global connection strength, and dynamic FC (*p* < 0.05). Both impaired NVC and increased FC correlate with age‐related decline in accuracy during the 2‐back task. These findings suggest that task‐related brain states in older adults require stronger functional connections to compensate for the attenuated NVC responses associated with working memory load.

## Graphical abstract

This study is designed to investigate the role of cerebrovascular regulation and functional brain networks in age‐related cognitive dysfunction using functional near‐infrared spectroscopy. The data indicate the association between impaired neurovascular coupling, increased functional connectivity and decreased working memory function in older adults. The findings suggest that cerebrovascular mechanisms play an important role in neurocognitive aging.

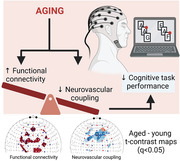

## 1 Introduction

Age‐related cognitive impairment is of great concern for the growing elderly population worldwide.^[^ ^1^ ^]^ Among the cognitive domains affected by aging,^[^ ^2^ ^]^ working memory impairment is of particular interest due to its early manifestation.^[^ ^3^ ^]^ Brain aging is a multifaceted process that is characterized by significant alterations in synaptic connectivity, particularly in the prefrontal cortex (PFC) and the hippocampus, despite an essentially preserved neuronal pool.^[^ ^4^ ^]^ Importantly, the cerebrovascular system and its function are also profoundly affected by aging.^[^ ^5^ ^]^ Age‐associated cerebrovascular pathological alterations range from arteriosclerosis^[^ ^6^ ^]^ and microvascular rarefaction to dysregulation of cerebral blood flow (CBF),^[^ ^7^ ^]^ all of which play a critical role in the genesis of vascular cognitive impairment (VCI), the second most common cause of dementia in older adults.^[^ ^8^ ^]^

Active neurons require continuous delivery of oxygen and nutrients via cerebral microcirculation which is ensured by a critical homeostatic mechanism termed as neurovascular coupling (NVC).^[^ ^9^ ^]^ Endothelial release of nitric oxide in the cerebral microvessels plays a key role in the prompt vasodilation underlying NVC tailored to the metabolically demanding brain tissue.^[^ ^9^, ^10^ ^]^ Preclinical studies established a causal relationship between age‐related NVC impairment and cognitive dysfunction.^[^ ^11^ ^]^ In animal models of aging, endothelium‐mediated NVC responses were found to be compromised^[^ ^9c^ ^]^ while anti‐aging interventions that rejuvenate cerebromicrovascular endothelial function improved both NVC responses and cognitive performance.^[^ ^11^, ^12^ ^]^ While a growing body of human studies documents the age‐related decline in NVC responses,^[^ ^13^ ^]^ the link between NVC dysfunction in the brain cortex and cognitive impairment in older adults has been relatively understudied.

Higher‐order cognitive processes recruit multiple, anatomically distinct brain regions and critically depend on physiological neuronal function and coordinated interaction within participating neural networks.^[^ ^14^ ^]^ Patterns of correlated neural activity define functional connectivity (FC) within the brain network of interest, which is thought to play a crucial role in cognitive processes and behavior.^[^ ^15^ ^]^ Several human studies revealed that resting‐state FC and its task‐induced change can predict cognitive performance.^[^ ^14^, ^16^ ^]^ Functional magnetic resonance (fMRI) studies of age‐related changes in functional brain networks found that in older adults FC is decreased within and increased between resting‐state networks, respectively.^[^ ^17^ ^]^ Task‐induced reorganization of functional brain networks was also found to be affected by aging^[^ ^18^ ^]^ and a significant association was established between the altered network properties and the performance during the working memory paradigm.^[^ ^19^ ^]^ While most of these studies utilized a static approach to characterize functional brain networks, it is possible that dynamic analysis of connectivity could better reflect the large‐scale neural mechanisms underlying age‐related cognitive dysfunction.^[^ ^20^ ^]^ Only a few recent studies examined functional networks in the brain cortex using functional near‐infrared spectroscopy (fNIRS) and reported age‐related differences in brain activation^[^ ^21^ ^]^ and measures of FC.^[^ ^21^, ^22^ ^]^ Contradicting the notion of impaired NVC, increased brain activity was observed in older adults^[^ ^22^, ^23^ ^]^ in response to cognitive stimulation which is associated with disrupted functional connectivity.^[^ ^22b,c^ ^]^ In contrast, other studies confirmed a generalized increase in FC,^[^ ^24^ ^]^ but such changes are also regarded as reorganization of brain networks.^[^ ^22^, ^25^ ^]^ Further research is needed in this emerging field to understand better the relationship between aging, cognitive performance, NVC, and FC in the brain cortex.

The present study aimed to determine the contribution of NVC impairment and alterations in FC to age‐related cognitive dysfunction. We hypothesized that both NVC and working memory function would be negatively affected by aging. We also hypothesized that aging would significantly impact the functional brain network topology in the frontal cortex, which is associated with age‐related alteration in NVC and predicts impaired cognitive performance. To test these hypotheses, we employed an fNIRS approach for examining NVC responses, as well as task‐related static and dynamic FC during an *n*‐back paradigm.

## 2 Results

### 2.1 Study Participants

The characteristics of the study population included in the analyses, medical conditions and baseline physiological measures are summarized in **Table** **1**. Data from nine participants were excluded from further analysis during data preprocessing due to poor channel quality as described in the methods section; thus, 21 young (33.23 ± 7.00 years of age) and 30 aged (76.13 ± 6.70 years of age) individuals were included in the final analyses. Body mass index, systolic and mean arterial blood pressure were significantly higher in the elderly group compared to the young group, while diastolic blood pressure values were statistically similar.

**Table 1:** Demographic, health conditions, medications and baseline physiological parameters of the study participants.

Characteristics	Young (*n* = 21)		Elderly (*n* = 30)	
	*n*	%	*N*	%
Sex				
Male	12	23.5	11	21.6
Female	9	17.6	19	37.3
Race				
American Indian or Alaska native	0	0	0	0
Asian	0	0	0	0
Black or Afro‐American	1	2	1	2.0
Native Hawaiian or other Pacific Islander	0	0	1	2.0
White or Caucasian	17	33.3	28	54.9
Asian Indian	3	5.9	0	0
Ethnicity				
Hispanic/Latino	0	0	1	2.0
non‐Hispanic / non‐Latino	21	41.2	29	56.9
Highest level of education				
Academic doctorate degree	6	11.8	3	5.9
Professional doctorate degree	3	5.9	0	0
Master degree	4	7.8	4	7.8
Bachelor degree	4	7.8	6	11.8
Other, no college or university degree	4	7.8	17	33.3
Handedness				
Left	1	2.0	0	0
Right	20	39.2	30	58.8
Active diseases				
Hypertension (controlled)	0	0	10	19.6
Other cardiovascular diseases	0	0	3	5.9
Diabetes mellitus (controlled)	0	0	2	3.9
Hypothyroidism	0	0	1	2.0
Other metabolic disorder	0	0	9	17.6
Psychiatric disease	0	0	8	15.7
Neurological disease	0	0	3	5.9
Diseases of bones, joints and muscle	2	3.9	13	25.5
Other medical conditions	3	5.9	12	23.5
Current smoker	1	2.0	0	0
Medications				
AT1‐receptor blocker	0	0	5	9.8
ACE‐inhibitor	0	0	5	9.8
β‐blocker	0	0	2	3.9
Ca‐antagonist	0	0	4	7.8
Diuretic	0	0	7	13.7
Statin	0	0	10	19.6
Estrogen supplement	0	0	2	3.9
Thyroid supplement	0	0	8	15.7
Other prescribed drugs	2	3.9	18	35.3

Baseline data	Mean	S.D.	Mean	S.D.
Systolic blood pressure* [mmHg]	115.7	16.7	126.1	12.6
Diastolic blood pressure [mmHg]	74.4	7.4	77.5	8.1
Mean arterial blood pressure* [mmHg]	88.2	8.2	93.7	8.4
Heart rate [beat per minute]	67.6	8.8	63.1	8.9
Body mass index* (BMI)	23.9	3.3	28.0	4.5

*: *p*<0.05

### 2.2 NVC‐Related Hemodynamic Responses Are Impaired in Older Adults

#### 2.2.1 Age‐Related Differences for the 2‐Back Task Condition

**Figure 1:**
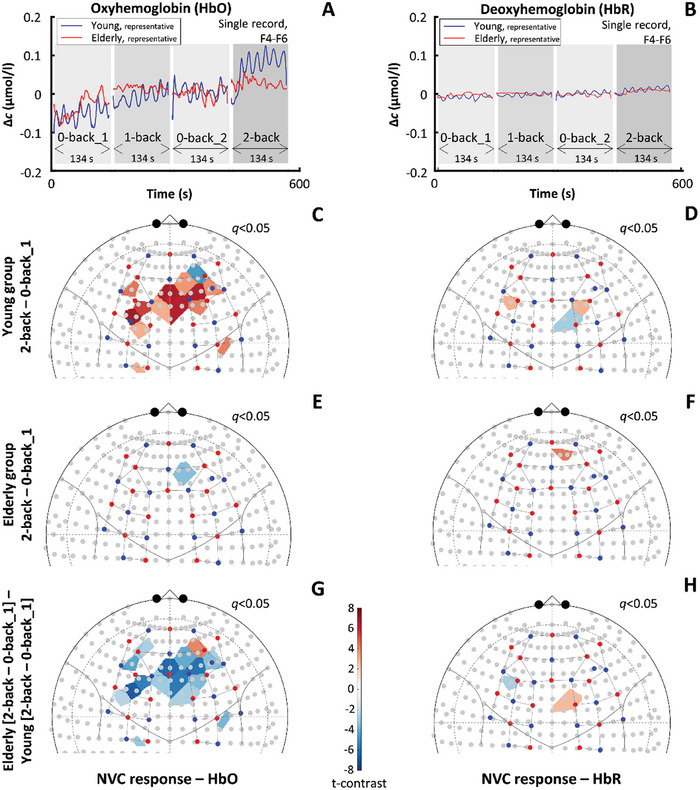
Impact of aging on neurovascular coupling (NVC) responses elicited by cognitive n‐back task in the prefrontal and motor brain cortices. All results are based on data from 21 young and 30 aged participants. In a representative young participant, A) the increase in oxyhemoglobin (HbO) during the 2‐back task exceeds the HbO rise in a representative aged participant. B) Shows the deoxyhemoglobin (HbR) changes; HbO and HbR records were obtained from the F4(source)‐F6(detector) channel belonging to the dorsolateral prefrontal cortex. Statistical contrast of HbO changes between the 2‐back and first 0‐back condition reveals significant NVC responses only for C) the young but not for E) the elderly group as indicated by the color‐coded t‐values with a cutoff at *q* < 0.05 (obtained after false discovery rate correction). For both age groups, D,F) localized changes can be seen for HbR changes between the same task conditions. Analyzing the effect of age on the contrast between 2‐back and first 0‐back condition revealed that HbO responses in prefrontal regions are significantly reduced (indicated by blue shades) in the aged group compared to G) the young group, but there is no notable difference for H) HbR responses. The standard channel positions in the current montage and the relation of the actual channel layout to the brain according to the 10–20 system were visualized by the AnalyzIR toolbox (Santosa, 2018); red and blue dots represent sources and detectors, respectively.

We compared the cerebral hemodynamic responses between age groups and across *n*‐back sessions by measuring oxyhemoglobin (HbO) and deoxyhemoglobin (HbR) in the brain cortex in response to cognitive challenge (**Figure** **1**). Representative traces show a pronounced increase in HbO during 2‐back compared to 0‐ and 1‐back conditions (Figure 1A), while HbR (Figure 1B) response remains largely unaltered across all three task difficulties. The output of the GLM revealed the impact of aging and the task condition on HbO and HbR responses during the entire ≈134 s long session, detailed descriptive statistics are provided as Supporting Information. To better distinguish age groups, we focused on the most challenging task (2‐back) and compared it with the baseline condition (first 0‐back task). Figure 1C–H map the false discovery rate (FDR)‐corrected *t*‐statistics for the [2‐back – 0‐back] contrast (*q* < 0.05). In young participants, a significant HbO increase was found between these conditions in the left and right dorsolateral prefrontal cortex (DLPFC) and in other prefrontal cortical areas (see red shaded areas in Figure 1C), as well as a localized HbO decrease in AF4 area. In contrast, HbO response was virtually absent for aged participants (Figure 1E). Accordingly, the [elderly–young] contrast map revealed significantly impaired HbO responses, particularly in the prefrontal cortex (PFC) including the DLPFC (see blue shaded areas in Figure 1G). In the case of HbR, contrast maps showed a much less extensive impact of task condition for the young (Figure 1D) and aged group (Figure 1F), compared to corresponding HbO statistics, as well as in [elderly‐young] contrast (Figure 1H). Thus, the impact of aging on HbR component of NVC‐related hemodynamic responses was neither significant except for two circumscribed regions in the left DLPFC and midline regions.

#### 2.2.2 The Effect of Task Difficulty on NVC‐Related Hemodynamic Responses

**Figure 2:**
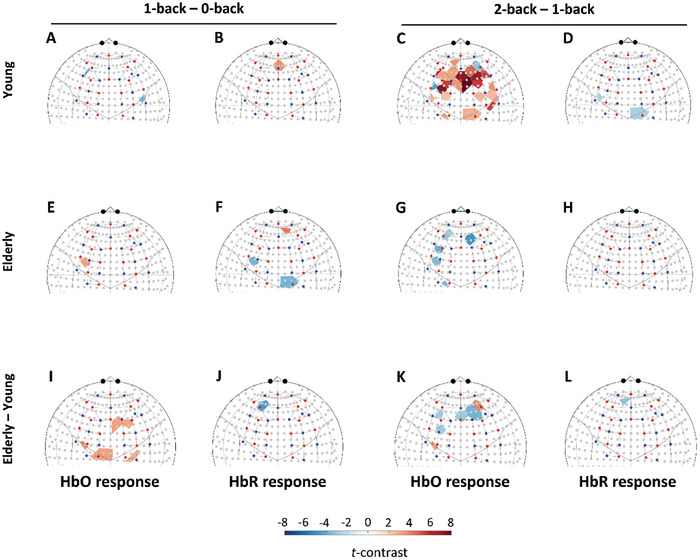
Effect of task difficulty on neurovascular coupling (NVC) responses elicited by cognitive n‐back task in the prefrontal and motor brain cortices of young and aged participants. All results are based on data from 21 young and 30 aged participants. Statistical contrast between the 1‐back and first 0‐back condition reveals focal HbO responses for A) the young and for E) the elderly group; HbR responses were also localized in B) the young and F) aged group. I,J) Map the age group‐related differences for HbO and HbR, respectively. Statistical contrast between the 2‐back and first 1‐back condition reveals a pronounced increase in HbO responses spreading across the prefrontal cortex for C) the young, while for the elderly, significant reductions can be observed in G) multiple brain regions. Changes in HbR responses can only be observed in one smaller area in D) the young group, while it is absent among H) older adults. I,J) Map the age group‐related differences for HbO and HbR, respectively. Colors refer to *t*‐statistics thresholded by *q* < 0.05 (obtained after false discovery rate correction). For both age groups, D,F) localized changes can be seen for HbR changes between the same condition. Analyzing the impact of age on the contrast between 2‐back and first 0‐back condition revealed that HbO responses in prefrontal regions are significantly reduced (indicated by blue shades) in the aged group compared to G) the young group, but there is no notable difference for H) HbR responses. K,L) Map the age group‐related differences for HbO and HbR, respectively. The standard channel positions in the current montage and the relation of the actual channel layout to the brain according to the 10–20 system were visualized by the AnalyzIR toolbox (Santosa, 2018); red and blue dots represent sources and detectors, respectively.

It is plausible that a stronger cognitive stimulus evokes a larger NVC‐related hemodynamic response. Thus, we assessed the effect of task difficulty on HbO and HbR responses by comparing the 1‐back condition to the reference (first) 0‐back and for the most challenging 2‐back condition to the less challenging 1‐back condition. Maps of *t*‐statistics for the 2‐back minus 1‐back condition and the 1‐back minus the first 0‐back condition are shown in **Figure** **2**. For the 1‐back minus 0‐back condition, we observed a significant HbO reduction in the young group (Figure 2A) accompanied by HbR increase (Figure 2B), and HbO increase in the elderly group (Figure 2E) accompanied by HbR changes (Figure 2F). For the 2‐back minus 1‐back condition, we observed a more pronounced and widespread HbO response, similar to the response seen for the 2‐back minus first 0‐back contrast, with additional involvement of motor cortices (Figure 2C). In contrast, HbO map for the aged group (Figure 2G) displayed a reduction in multiple areas. Simultaneously, HbR responses were reduced in the young group (Figure 2D) and unaltered in the aged group (Figure 2H). Elderly group demonstrated significantly higher HbO response in the motor cortex during 1‐back condition (Figure 2I) and significantly lower HbO responses in the PFC during 2‐back condition (Figure 2K), while HbR responses were unaltered both in case of 1‐back (Figure 2J) and 2‐back condition (Figure 2L). The impact of sex on NVC and its age‐related differences are reported in Figure S1 (Supporting Information). Since the distribution of education level was not equal between the young and old group (*p* = 0.005, χ^2^‐test for their association), it might have influenced the cognitive‐task induced NVC responses in an age‐specific manner. Although HbO maps suggest a significant effect of education level, the group effect is much smaller compared to age effect according to size of affected cortical areas (Figure S3, Supporting Information).

#### 2.2.3 Dynamics of NVC Responses in the Frontal Brain Cortex

**Figure 3:**
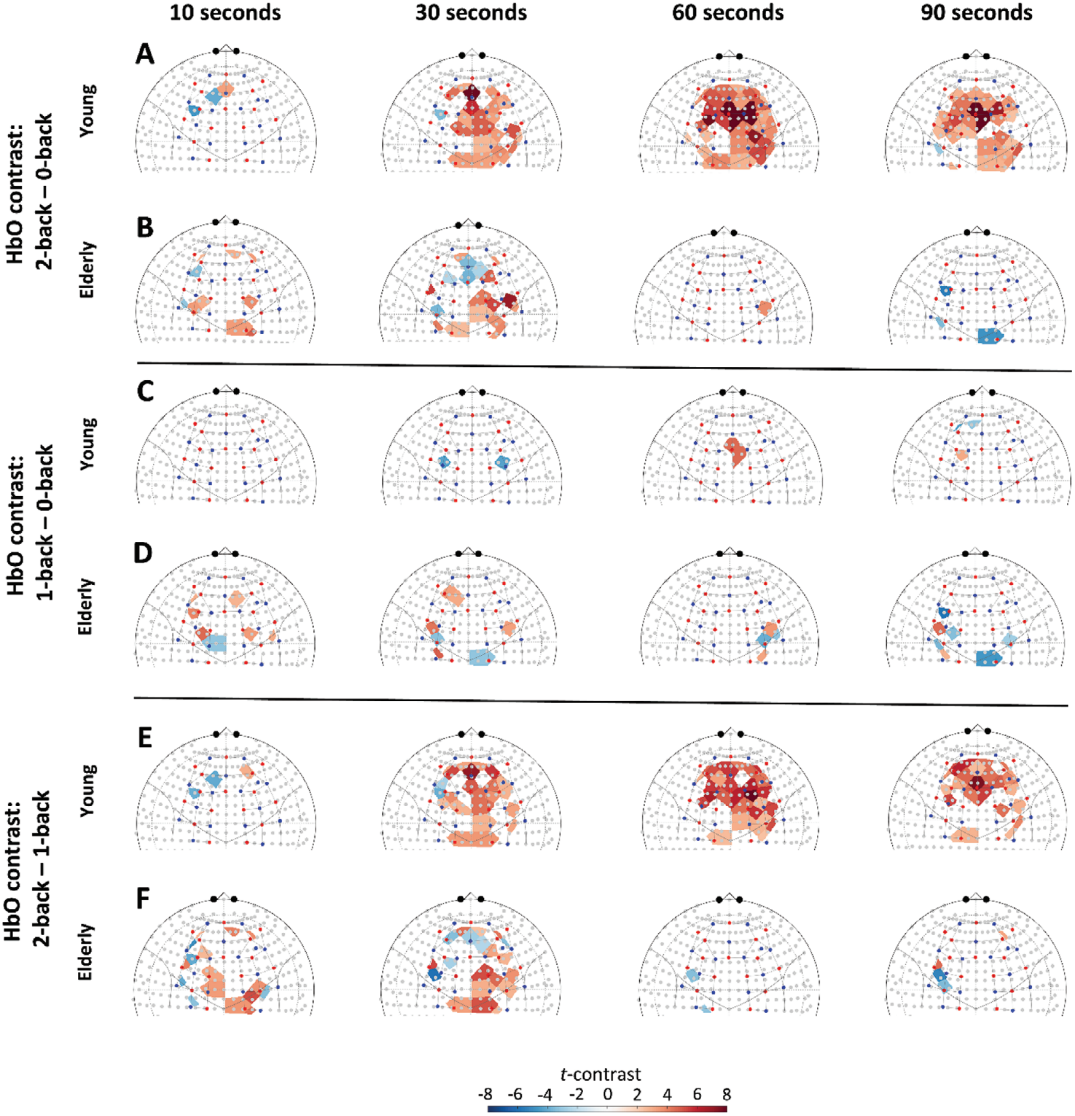
Impact of aging and task difficulty on the dynamics of neurovascular coupling (NVC) responses evoked by n‐back paradigm. All results are based on data from 21 young and 30 aged participants. Group‐level analysis of NVC responses was assessed by varying the length of time considered after the beginning of each session (stimulus duration): 10 s, 30 s, 60 s, and 90 s young group. Statistical contrast of HbO changes between 2‐back and first 0‐back conditions are shown separately for A) the young and for B) the elderly group. For the same stimulus durations, HbO maps were obtained displaying the 1‐back minus first 0‐back contrast in C) the young and D) elderly group and displaying the 2‐back minus 1‐back contrast in E) the young and F) elderly group. Please note the marked NVC responses in the young group after 30 s concerning wide regions in the frontal cortex that significantly exceeds the milder NVC response in the elderly when the 2‐back session was compared to the 1‐ or 0‐back session. Corresponding HbO contrast maps after 60 and 90 s suggest that hemodynamic response is maintained in the young group for a longer period while it fades in the elderly group, even reaching a significant decline in regions of the motor cortex. The standard channel positions in the current montage and the relation of the actual channel layout to the brain were visualized by the AnalyzIR toolbox (Santosa, 2018). For further details, see the main text.

The results for dynamic analysis of NVC‐related hemodynamic responses for HbO are shown in **Figure** **3**. In the case of 2‐back minus first 0‐back contrast, our data showed the development of a pronounced HbO response in the young group (Figure 3A), which was present after 10 s from the beginning of cognitive *n*‐back task, became widespread across the frontal cortex after 30 s, and persisted for 60 s and 90 s until the end of the stimulation. In contrast, HbO responses in the elderly group (Figure 3B) were attenuated compared to the young group. In the case of 1‐back minus first 0‐back contrast, we observed much smaller responses in both young (Figure 3C) and aged (Figure 3D) groups, with a fluctuating dynamics not exhibiting a clear directionality of change in HbO. Interestingly, the older adults showed higher cortical hemodynamic responses compared to young adults. HbO maps showed the development of a pronounced response for the 2‐back minus 1‐back in the young group (Figure 3E), which were present after 10 s from the beginning of stimulation, became widespread across the frontal cortex after 30 s, and persisted for 60 s and 90 s. In contrast, HbO responses in the elderly group (Figure 3F) were attenuated compared to the young group, as significant increases were less pronounced and localized to smaller areas of the frontal cortex.

Data for HbR responses are not shown as it was not affected by aging or task condition as described and shown above.

### 2.3 Functional Connectivity Is Increased in Aged Participants

#### 2.3.1 FC and Local Network Measures in the Frontal Brain Cortex during Cognitive Stimulation

**Figure 4:**
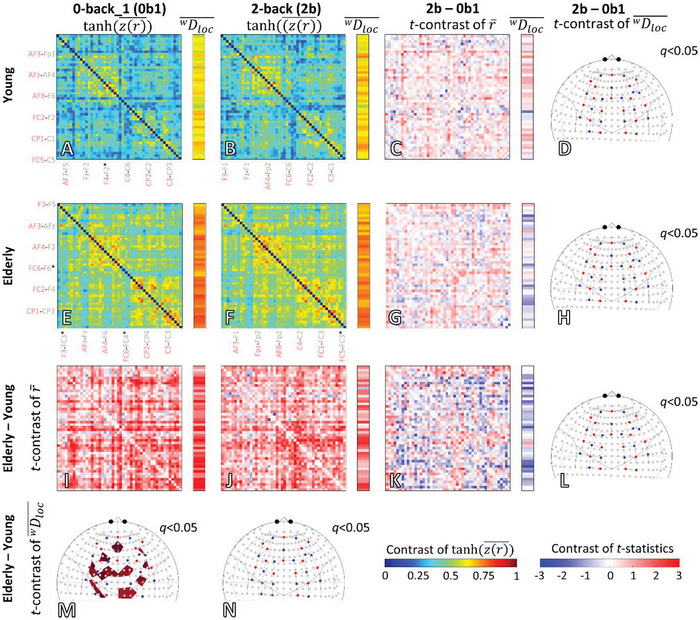
Statistical effect of age and task condition on local functional connectivity (FC) and local network metrics. Task effects are shown in rows; age effects are shown in columns. Color‐coded grand averages of Fisher *z*‐transformed Pearson‐adjacency matrices and network metrics are displayed for age groups and task conditions: A) young 0‐back_1, B) young 2‐back, E) elderly 0‐back_1 and F) elderly 2‐back. Each value in the average matrix represents the inverse Fisher‐transform—obtained by the *a*tan*h* function—of the mean of Fisher‐transformed *r*, referring to an individual connection between the corresponding channels. The contrast between 0‐ and 2‐back conditions is displayed as a color‐coded matrix of *t*‐statistics obtained from pairwise comparisons in C) the young group (*n* = 21,) and in G) the elderly group (*n* = 30,). Similarly, the FC of the young and elderly groups were compared by calculating the statistical contrast (unpaired t‐statistics) between adjacency matrices for the 0‐back_1 (0b1) and 2‐back (2b) conditions. Statistical contrast maps obtained for I) 0b1 and J) 2b conditions indicate higher FC in the elderly group. In addition, a group‐ and task‐specific contrast was determined to assess the impact of aging on the channel‐wise connectivity response during 2b with respect to K) 0b1: [(elderly 2b—elderly 0b1)–(young 2b—young 0b1)]. For each pair of the channel, the mean weighted local node degrees ( 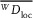) are obtained as row‐wise averages of $\bar{r}$ values representing connection strengths. Following a similar statistical approach for characterizing the age‐ and group‐specific changes, the *t*‐contrast of 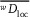 are also shown on the right to the corresponding color‐coded connection matrix. Finally, significance thresholded (*q* < 0.05, false discovery rate corrected) *t*‐contrasts of 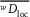 are mapped into the brain cortex for each statistical comparison: D) young 2b—young 0b1, H) elderly 2b—elderly 0b1, M) elderly 0b1—young 0b1, N) elderly 2b—young 2b, and L) [(elderly 2b—elderly 0b1)–(young 2b—young 0b1)]. Group‐ and task‐specific average adjacency matrices are color‐coded between 0 and 1; several channels are indicated below and to the left. Matrices and brain maps of statistical *t*‐contrasts are scaled in the [‐3 3] range.

Local measures of FC (surrogate‐thresholded Pearson‐correlation coefficient, $r_{ij}^{*}$) and mean weighted local node degree 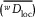, a local network metrics calculated for each fNIRS channel are presented in **Figure** **4** as group averages. To compare the $r_{ij}^{*}$ values between different age groups and task conditions, the averaging step was carried out after obtaining a normally distributed sample as$atanh(\bar{z(r_{ij}^{*})})$, where *z*() and *atanh*() refer to *z*‐transform and its inverse, respectively. Then statistical *t*‐contrast was calculated for such local measures of FC and for 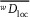 of the nodes— representing a sampled brain region—in the assessed functional network in the frontal cortex. Figure 4A–D displays the average connection matrices and the mean of 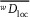 values in the young group, along with *t*‐statistics for the contrast between 2‐back versus first 0‐back conditions. The corresponding group averages and statistics in the elderly are visualized in Figure 4E–H and indicate that task condition did not significantly affect local connectivity in either age group. Local connection strengths and network measures demonstrated a widespread increase in the elderly group for both the first 0‐back and 2‐back conditions (Figure 4I–J), which was significant for the first 0‐back condition. Similar but non‐significant age‐related changes can be observed for the 1‐back condition (data not shown). However, task‐induced changes of age‐related increase in FC are characterized by higher 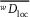 were insignificant, as reflected by the elderly vs. young differential connection matrices (Figure 4K) and maps (Figure 4L). Taken together, we found a significant age effect for the first 0‐back condition in particular, while task difficulty did not influence measures of FC local network metrics.

#### 2.3.2 Global Network Measures of Static FC in the Frontal Brain Cortex

**Figure 5:**
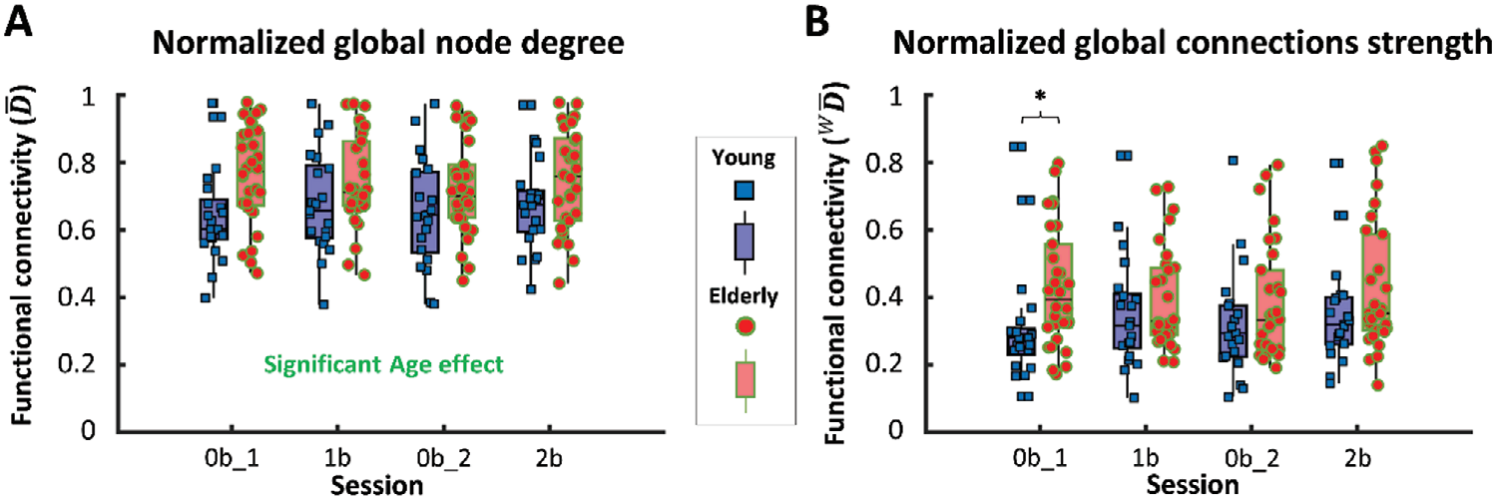
Age‐related changes in global network metrics characterizing static functional connectivity (FC) in the frontal cortex during sessions of n‐back task. Individual global (i.e., referring to the whole frontal cortex) network metrics values are shown in blue squares (young group, *n* = 21) or red dots (elderly group, *n* = 30) separately for 0‐back_1 (0b1), 1‐back (1b), 0‐back_2 (0b2) and 2‐back (2b) sessions. Corresponding median values and interquartile ranges are displayed as purple (young group) or pink (elderly group) boxplots. A) Normalized global node degree ( $\bar{D}$) is significantly higher in the elderly group throughout all task sessions, as indicated by GLM and confirmed by the Tukey post‐hoc test (*p* = 0.0282). B) Normalized global connection strength ( $W\bar{D}$) differs significantly only for 0b1 session as indicated by the Mann‐Whitney test (*p* = 0.006).

**Figure** **5** shows the effect age and task on normalized node degree $\left(\bar{D}\right)$ and connection strength 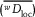. In agreement with the results for local FC, we found a significant effect of age (GLM: *p* = 0.0281 for the group effect) on $\bar{D}$, a global network measure reflecting the average number of connections normalized to the number of all possible connections (Figure 5A). The observed age‐related difference in normalized node degree values were confirmed by Tukey's post hoc test (*p* = 0.0282, *F*(1,49) = 5.121), and $\bar{D}$ was higher in the elderly group independently from task condition (GLM: *F*(3,147) = 1.24 and *p* = 0.297 for the main effect; *F*(3,147) = 1.567 and *p* = 0.200 and for the interaction between age and task). The median of corresponding weighted network measure values was significantly higher in the aged group only during the 0‐back task (Figure 5B), indicating that binarization enhances the age‐related differences in connectivity. The task effect was not significant on $\bar{D}$ (GLM: *p* = 0.164), but on 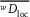 for the whole study population (*p* = 0.008, Friedman‐test) and for the elderly group (*p* = 0.019, Friedman‐test). Regarding $\bar{D}$, the effect of age remained after adjusting for sex or education, while it was neither significantly different (Tukey's *p* = 0.111) between male and female participants (see Figure S2, Supporting Information); nor between participants with different level of education (*p* = 0.478), see Figure S4 (Supporting Information).

#### 2.3.3 Dynamics of FC in the Frontal Brain Cortex

Results of statistical analysis comparing young and aged groups for each *n*‐back session are reported in Table S4 and Figure S3 (Supporting Information).

### 2.4 Working Memory Is Impaired in the Elderly Group

**Figure 6:**
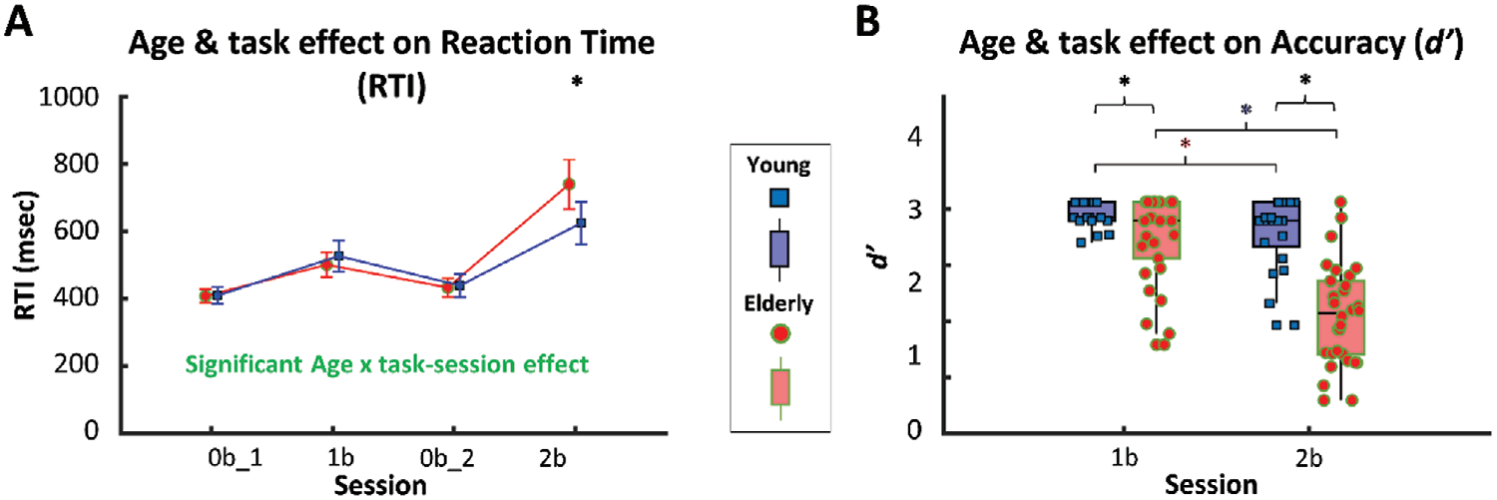
Age‐related changes in cognitive outcome measures of n‐back paradigm. A) Mean, and confidence intervals are shown for behavioral variables that are displayed as blue squares (young group, *n* = 21) or red dots (elderly group, *n* = 30) separately for 0‐back_1 (0b1), 1‐back (1b), 0‐back_2 (0b2) and 2‐back (2b) sessions. The interaction of the task session and age group was found significant, as indicated by GLM (*p* = 0.0029) and confirmed by Newman‐Keuls post hoc test (*p <* 0.0001). B) Individual values of behavioral variables are displayed as blue squares (young group) or red dots (elderly group) separately for 0b1, 1b, 0b2 and 2b sessions. Corresponding median values and interquartile ranges are displayed as purple (young group) or pink (elderly group) boxplots. Accuracy was characterized by *d’* (see the formula in the main text). Significant differences were found between the young and elderly groups both for 1b (*p* = 0.027, unpaired t‐test) and 2b (*p* < 0.0001, Mann‐Whitney U test) conditions. According to Wilcoxon‐test, *d’* was significantly decreased for 2b session compared to 1b session in both young (*p =* 0.0442, purple asterisk) and elderly groups (*p<*0.0001, pink asterisk).

We found significant differences both in reaction time (RTI) and accuracy (*d’*) between young and aged participants (**Figure** **6**). Figure 6A displays the changes in reaction time (RT) in the young and elderly groups across all task conditions. GLM revealed significant task effect (*F*(3,147) = 83.56, *p* < 0.0001) and interaction (*F*(3,147) = 5.654, *p* = 0.0029) between task condition and age group, both confirmed by Newman‐Keuls post‐hoc test (*p* < 0.0001). As expected, RTI increased (*p* < 0.0001 for all comparisons with different *n*) with higher *n* in both age groups due to the greater working memory load. Reaction times were significantly higher in the elderly group only in case of 2‐back task condition (*p* < 0.05, unpaired t‐test).

Regarding working memory performance, Mann‐Whitney test revealed a significant impact of aging on *d’* for the 2‐back task, but not for the 1‐back task (Figure 6B). As expected, we found that participants performed poorer in case of 2‐back compared to 1‐back task condition for both age groups (*p* < 0.05, Wilcoxon‐test).

### 2.5 Impaired NVC and Increased FC Associate with Decreased Cognitive Performance

**Figure 7:**
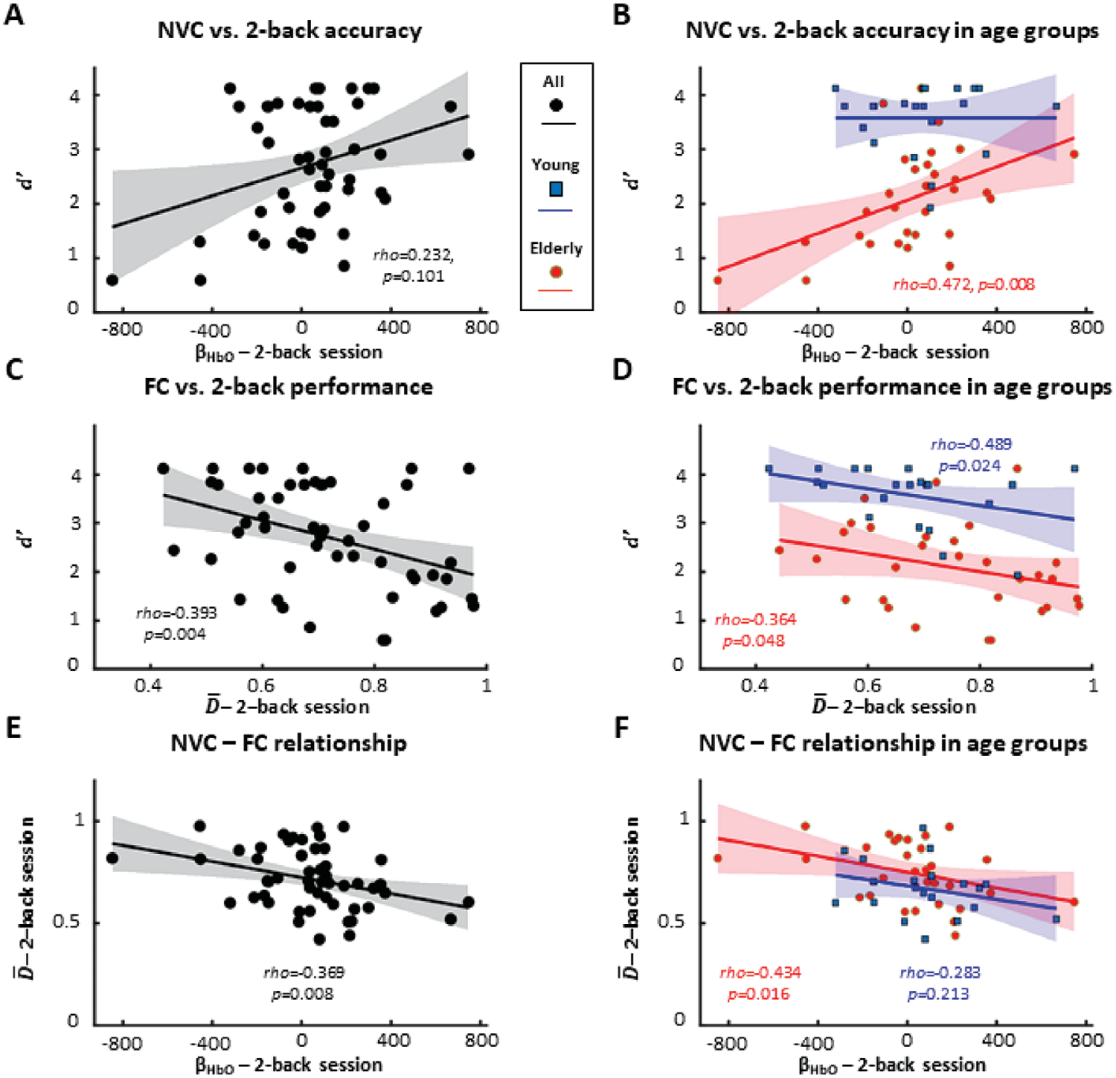
Impaired neurovascular coupling responses and increased functional connectivity associates with decreased cognitive performance in aged persons. A,C,E) Black dots, line and shaded area mark individual values corresponding to the 2‐back session, linear regression line and its confidence interval, respectively. B,D,F)Red/blue lines and light red/light blue shaded area mark linear regression lines and their confidence intervals, respectively; blue squares (young group, *n* = 21) and red dots (elderly group, *n* = 30) depict individual values. A) Accuracy (*d’*) characterizing cognitive performance during the 2‐back session is plotted against regression coefficient (*β*_HbO_) characterizing neurovascular coupling (NVC) response for oxyhemoglobin (HbO). For the whole study population, *β*_HbO_ and *d’* are inversely related; however, their rank correlation did not reach the significance level (Spearman's *p* > 0.05). B) The relationship between *d’* and β_HbO_ was also assessed in each age group separately. Individual values correspond to 2‐back session and are displayed as blue squares (young group, *n* = 21) or red dots (elderly group, *n* = 30). Rank correlation analysis reveals a significant positive association only for the elderly groups between β_HbO_ and *d’* (Spearman's rho = 0.472). C) Two‐back accuracy is plotted against the global normalized node degree ( $\bar{D}$) characterizing static FC. Analysis of rank correlation reveals a significant negative association between $\bar{D}$ and *d’* (Spearman's *rho* = ‐0.392) in the whole study population. D) The relationship between $\bar{D}$ and *d’* was also assessed in each age group separately. Analysis of rank correlation between $\bar{D}$ and *d’* reveals a significant negative association both for the young (Spearman's *rho* = ‐0.489) and for the elderly group (Spearman's *r* = ‐0.364). E), characterizing FC is plotted against β_HbO_, characterizing NVC for HbO. Analysis of rank correlation reveals a significant negative association between *β*_HbO_ and $\bar{D}$ (Spearman's rho = ‐0.369). (F) The relationship between $\bar{D}$ and *β*_HbO_ was also assessed in each age group separately. Analysis of rank correlation reveals a significant negative association only for the elderly group between *β*_HbO_ and $\bar{D}$ (Spearman's rho = ‐0.434).

Correlations between fNIRS‐based parameters and cognitive performance are shown in **Figure** **7**. First, we found that impaired NVC significantly correlates with lower 2‐back accuracy (Figure 7A,B). The strength of the relationship between the ranks was significant in the elderly group (rho = 0.472, *p* = 0.008) but not in the young group (rho = 0.076, *p* = 0.744), featuring smaller variability, particularly in *d’*. Inclusion of young group also weakens the relationship between *d’*_2b_ and the regression coefficient β_HbO_ for the whole study population reflected by non‐significant rank correlation. These results establish a direct link between reduced hemodynamic response in the frontal cortex evoked by cognitive stimuli and poorer performance during the same paradigm. Although we were not able to examine the effect of age and β_HbO_ on *d’* in the same statistical model due to violation of assumptions, the strength of such relationship is clearly different between the young and elderly groups.

Second, we found that increased FC significantly correlates with lower 2‐back accuracy as depicted in Figure 7C,D. Rank correlation analyses revealed a significant inverse relationship for all participants (rho = ‐0.392, *p* = 0.004) and for both the young (rho = ‐0.489, *p* = 0.024) and elderly group (rho = ‐0.364, *p* = 0.048). Our data thus demonstrate a strong association between global network metrics characterizing the assessed FC of the frontal lobe and cognitive performance during the *n*‐back paradigm, which relationship is independent of age group.

Finally, we assessed whether the key fNIRS parameters sensitive to age group effect were interrelated, although their correlation with 2‐back accuracy indirectly implies that *β*_HbO_ and $\bar{D}$, physiological parameters referring to the whole frontal cortex are not independent. Figure 7E,F confirms the expected inverse relationship between *β*_HbO_ and $\bar{D}$, whose correlation is significant for all participants (rho = ‐0.369, *p* = 0.007) and in the elderly group (rho = ‐0.434, *p* = 0.048), but not in the young group (rho = ‐0.283, *p* = 0.213). These results capture the dependencies between correlated hemodynamics and evoked hemodynamic responses under the greatest working memory load.

## 3 Discussion

In this cross‐sectional observational study, we investigated the effects of aging on cognitive function, NVC responses and functional brain networks in the frontal cortex. To test our hypothesis that aging alters cognitive function, NVC, and FC, we compared healthy young adults and community‐dwelling older adults using an fNIRS approach during a working memory *n*‐back paradigm. We found that older adults exhibited worsened cognitive performance, decreased prefrontal NVC responses under the highest working memory load, and increased global metrics of frontal brain networks during the entire *n*‐back paradigm. Additionally, we tested the hypothesis that age‐related attenuation of NVC and increased FC under working memory load predict poor cognitive performance. We observed that age‐related impairment of NVC responses and an increased number of functional connections were associated with lower *n*‐back accuracy. Our results suggest that frontal brain networks compensate for impaired NVC responses by recruiting additional functional connections, in line with the compensation‐related utilization of neural circuitry hypothesis (CRUNCH).^[^ ^26^ ^]^ These findings contribute to a better understanding of how aging affects brain function and have potential implications for developing interventions to support healthy aging.

Our cognitive assessment protocol was able to detect age‐related changes in working memory function, which is often affected early in the aging process compared to other cognitive domains,^[^ ^27^ ^]^ and is linked to areas in the PFC, particularly the left DLPFC.^[^ ^28^ ^]^ However, more widespread involvement of the PFC is typical in aging, as reported in the literature.^[^ ^19^, ^29^ ^]^ Consistent with previous research, our results showed that older adults performed less accurately and responded more slowly than the young group, with longer reaction times (Figure 6A) and reduced accuracy (Figure 6B) during the most challenging *n*‐back task condition.^[^ ^30^ ^]^ These findings provide further evidence for the impact of aging on cognitive function, particularly working memory, and underscore the importance of investigating the underlying neural mechanisms to develop effective interventions for promoting healthy aging.

The fNIRS method has been used in numerous studies of working memory^[^ ^31^ ^]^ since the frontal cortex associated with this cognitive domain is well accessible to NIRS measurements. In this study, we evaluated NVC responses by comparing the 2‐back (Figure 1) and 1‐back (Figure 2) task conditions with the 0‐back task condition. For the young group, significant widespread HbO responses were found in the PFC during the 2‐back task (Figure 1C). In contrast, in the elderly group, HbO increase was absent in the frontal cortex during this session (Figure 1E). The spatially extended response can be attributed to local and upstream vasodilation in the cerebrocortical circulation as, in young adults, we expect significant neural activities only in DLPFC areas specific to working memory function.^[^ ^28^ ^]^ However, the employed paradigm may activate other prefrontal brain regions as well. A smaller cognitive workload brings about smaller NVC responses in principle, as we have shown previously.^[^ ^32^ ^]^ However, we only observed this pattern in the young group, while in the aged group, brain activation was higher during the 1‐back task. This points to age‐related differences in neural activity elicited by n‐back tasks of the same difficulty, in contrast to our initial assumption.

Regarding task‐condition‐related differences, the 2‐back task evoked reduced HbO responses compared to the 1‐back task only in the elderly group, which could be attributed to saturation of neural activity at a smaller cognitive workload. Such “neurovascular fatigue” can also be implicated since the 2‐back task is administered at the end of the *n*‐back paradigm, and dynamic analysis reveals attenuated HbO responses that cease more quickly in older adults (Figure 4). Neuroimaging studies have shown that in older adults free of neuropsychiatric diseases, the increase in prefrontal neural activity is greater in response to cognitive stimuli than in young adults.^[^ ^33^ ^]^ This observation was confirmed in fNIRS studies of aging using paradigms for verbal working memory^[^ ^16b^ ^]^ and executive function.^[^ ^22c^ ^]^ Assuming that such age‐related differences in neural activities are also present in the obtained HbO maps, it indeed confirms NVC impairment in the elderly group as their hemodynamic responses are attenuated despite the higher neural activity.

Taken together, our results indicate impaired prefrontal NVC responses in the aged group induced by cognitive n‐back tasks. These findings align with the literature on age‐related impairment of NVC responses in animal models of aging^[^ ^9c–e^ ^]^ and in human studies under resting‐ and task states.^[^ ^13c^ ^]^ Considering the mechanistic inferences, impaired NVC limits the ability of cognitively intact older adults to allocate sufficient resources required by increasing working memory load and implies age‐related changes in the underlying neurovascular unit. Preclinical studies demonstrated that the mechanisms underlying age‐related impairment of NVC responses include microvascular endothelial dysfunction^[^ ^7^ ^]^ due to increased oxidative stress, cellular mitochondrial dysfunction and acquisition of a senescent phenotype. In a largely overlapping cohort from the same study, we showed that peripheral vascular function is impaired in older adults suggesting an age‐related endothelial dysfunction. Since endothelial dysfunction underlies hypertension^[^ ^34^ ^]^ which in turn promotes impaired cerebromicrovascular function,^[^ ^35^ ^]^ it likely accounts for the relationship between age‐related changes in blood pressure and reduced NVC responses. Since intact NVC responses critically depend on NO released by the endothelium,^[^ ^10^ ^]^ our findings potentially implicate impaired cerebrovascular endothelial function.

To characterize the functional brain networks in the frontal cortex of our study participants, we conducted static and dynamic analyses of FC using significant Pearson‐coefficients. Our results showed that older adults had a higher global node degree, indicating a greater number of statistically interdependent hemodynamics related to neural activity (Figure 5A). However, global connection strength was only able to differentiate between the young and aged group for the first 0‐back condition (Figure 5B), which was consistent with local connection strength. Notably, the latter was significantly higher among the elderly in numerous frontal brain regions (Figure 4). Additionally, dynamic analysis of global connection strengths demonstrated a denser functional network in the frontal cortex of the aged participants that persisted throughout all *n*‐back task sessions (Figure S4, Supporting Information). Importantly, our methodological approach supports the notion that age‐related differences in FC do not depend on the chosen duration of the analytical time window.

In our study, we found that only the mean and variance of $w\bar{D}$ calculated for the 10‐ and 30 s time windows were significantly higher in the aged group compared to the young group for all task sessions (Figure S4, Supporting Information), indicating that faster dynamics of global network properties are more sensitive to the aging effect. We did not observe spatially concentrated changes in frontal brain network reorganization that could have been captured by local graph theoretical parameters emphasizing the role of brain regions particularly associated with the task. Ultimately, these findings correspond to the age‐related increase in resting‐ and task‐state FC captured by fMRI,^[^ ^17^ ^]^ albeit at a much lower spatial resolution and restricted to the brain cortex.

Previous fNIRS research have also revealed the impact of age on FC of the frontal cortex.^[^ ^21^, ^22^ ^]^ However, the observed age‐related alterations varied across studies which may be attributed to differences in task, channel positions, or analytical approach. Specifically, in our study, we utilized the *n*‐back paradigm to probe working memory function, whereas other studies utilized the Stroop test to assess executive function.^[^ ^22c^ ^]^ Despite repeating the analytical pipeline of these previous studies, our elderly group still exhibited higher FC, suggesting that differences in outcomes can be ascribed to the different paradigms utilized in these studies.

Given that CBSI yields a hemodynamic signal with an enhanced representation of neural activity,^[^ ^36^ ^]^ the age‐related alterations we observed in functional brain networks and NVC responses represent fundamentally different aspects of adaptation to mental workload. Specifically, our results suggest that functional brain networks in the frontal cortex become less efficient with aging under cognitive workload as more connections are recruited for the same mental state. This is consistent with the notion that the age‐related increase in FC serves as a compensatory mechanism to support adaptation to cognitive demands in older adults.^[^ ^19^, ^24^, ^26^ ^]^ Interestingly, our findings are also in line with previous studies that reported a more pronounced increase in connectivity in older patients with mild cognitive impairment.^[^ ^24^, ^25^ ^]^

NVC plays a crucial role in maintaining neuronal homeostasis by removing metabolic waste and supplying nutrients and glucose. The age‐related decline in NVC responses observed in our study's older participants could potentially impair cognitive functions that depend on stable neuronal activities. Our findings showed that the age‐related impairment of HbO response during the 2‐back task in the frontal cortex was associated with decreased accuracy in the elderly group (Figure 7B). In contrast, HbO response of the frontal cortex did not correlate with 2‐back accuracy in the young group, probably because this condition did not challenge them (Figure 7A). Previous preclinical studies have shown that the magnitude of NVC responses correlates with cognitive performance and that pharmacological treatments that improve NVC responses in old mice restore cognitive function to youthful levels.^[^ ^11a,c^ ^]^ In a human study using transcranial Doppler sonography, Sorond et al. found a significant correlation between middle cerebral artery blood flow velocity and *n*‐back performance.^[^ ^37^ ^]^ These findings suggest that impaired NVC likely contributes to age‐related impairment of cognitive function in humans as well. Conversely, our pilot cross‐sectional data from older patients with peripheral artery disease suggest that further impairment of NVC could exacerbate cognitive decline.^[^ ^38^ ^]^ In summary, our study identified impairment of NVC as a key vascular mechanism underlying cognitive aging that may contribute to the development of VCI.

The cognitive processes that underlie working memory rely on the functional interaction between neural assemblies, which suggests a link between FC and cognitive performance. Our study found that increased connection strengths were associated with decreased accuracy (Figure 7C,D), indicating that cognitive processes are impaired or do not benefit from the recruitment of functional connections. However, interpreting such relationships is complicated for several reasons. First, aging has a distinct effect on connectivity within and between resting‐state networks,^[^ ^17b,c^ ^]^ whose investigation is limited by our fNIRS setup probing 48 regions in the frontal brain cortex. Second, the brain exhibits high activity in the resting state,^[^ ^39^ ^]^ and transition into the task state typically implicates reorganization of functional connections associated with the applied paradigm. Here, we interpret functional brain networks with increased connectivity as being less “effective” due to higher wiring cost. Several fMRI studies have found a significant relationship between cognitive performance and network metrics, the nature of which essentially depended on the brain regions of interest.^[^ ^17^, ^40^ ^]^ In an EEG study, Kaposzta et al. also found that decreased connectivity correlated with better 3‐back performance.^[^ ^16c^ ^]^ Notably, the *n*‐back task difficulty level did not have a significant effect on global network metrics (Figure 5), but it did affect the reorganization of brain networks, which was heterogeneously localized in our study population. We observe this transition right after the first 0‐back task, which is characterized by the greatest age‐related difference (Figure 4M,N) and likely most similar to the preceding resting‐state. This reorganization of the frontal brain network is rather dominated by the recruitment (increase in FC) in the young group, while pruning of functional connections in the elderly group. Both processes are important for the adaptation to higher mental challenges that require task‐specific functional connections but can be disturbed by task‐irrelevant functional connections. This reorganization compensates for working memory load but does not affect performance at lower loads in any groups. Moreover, the reorganization of functional connections is insufficient to maintain accuracy at the highest level of mental workload. Therefore, we identified a measure of physiological brain function (“brain health”) based on the functional connectivity within the frontal brain network that predicts cognitive performance.

Given the significant association between cognitive performance and fNIRS‐based parameters, standardized regression coefficients for NVC response and global functional connection strength are also significantly correlated (Figure 7). Their inverse relationship is significant only in elderly group, supporting CRUNCH as likely explanation. Recent review highlighted weaknesses and limitations of CRUNCH,^[^ ^41^ ^]^ but a plethora of neuroimaging studies put it forward as an explanation for age‐related cognitive dysfunction.^[^ ^3^, ^16^, ^19^, ^33^ ^]^

Here we propose CRUNCH as a possible explanation for the changes observed in NVC and FC during the working memory task. Our findings shed light on the vascular mechanisms underlying healthy cognitive aging, revealing that intact NVC in the frontal cortex plays a critical role in higher cognitive performance in aging. Moreover, age‐related impairment of NVC responses is accompanied by insufficient reorganization of frontal brain networks, which is characterized by higher FC, likely due to compensation and becomes inadequate at a higher cognitive workload. These changes are more pronounced in patients with MCI,^[^ ^24^, ^25^ ^]^ and they may contribute to the genesis of VCI as well,^[^ ^8^ ^]^ as long as the primary cause of cognitive decline cannot be attributed to significant neuronal death or dysfunction. Overall, our study highlights the importance of vascular factors in cognitive aging and provides insights into the neural mechanisms underlying cognitive decline in aging and related disorders.

Considering the study population, our results can only be generalized to white people as other races were underrepresented in our study population (see Table 1) and their exclusion from the analyses did not affect any of the main results reported in this paper. Another remarkable difference between age group is the unbalanced presence of comorbidities between age groups. However, all comorbidities have been adequately controlled at the time of assessments. Our data revealed small (mostly insignificant) effect of sex or education compared to age‐related changes, which may be more prominent with a larger study population with equally represented categories. Another limitation is the lack of stratification by body mass index (BMI). Obesity is associated with microvascular endothelial dysfunction and impaired NVC responses.^[^ ^42^ ^]^ However, our data from older adults^[^ ^42^ ^]^ and current literature show that the impact of obesity is less significant compared to aging effects. Although participants with uncontrolled hypertension were not eligible for this study, differences in blood pressure could also have affected the observed fNIRS parameters. Since higher blood pressure, which is more prevalent in older adults, promotes endothelial dysfunction^[^ ^34^ ^]^ and associated with impairment of endothelium‐dependent NVC responses,^[^ ^35^ ^]^ its mechanism of action largely overlaps with the mechanism of aging underlying our findings. Hence, any factors promoting endothelial dysfunction cannot be regarded as a confounding variable, studying their role is beyond the scope of this study.

Our fNIRS approach captures hemodynamic responses which may reflect either altered NVC responses, systemic vascular effects or different neural activities.^[^ ^43^ ^]^ The contributions of varying degrees of neuronal activation and systemic hemodynamics could be assessed by independent simultaneous EEG measurements and blood pressure measurements, respectively, which were not carried out in this study. However, as discussed above, age‐related changes in neural activity are unlikely to explain the alterations in elicited cortical hemodynamic responses. To draw causal inferences, future human studies focusing on age‐related cognitive dysfunction should address the limitations mentioned above. Longitudinal studies, preferably with a decade or longer follow‐up period, are highly needed. In our cross‐sectional study, we examined potential markers of cerebrovascular aging whose predictive value regarding cognitive deterioration is worth investigating in future studies. Further research in this field will help to better elucidate the mechanisms of neurocognitive aging and its relationship with cerebrovascular impairment.

In summary, the present fNIRS study sheds light on the impact of aging on cognition, NVC, and functional brain networks in the frontal cortex. Our findings suggest that NVC responses are diminished in older adults during a working memory task and that this decline is associated with poorer working memory performance. Furthermore, we observed that aging is characterized by increased task‐state functional connectivity, which is inversely related to cognitive performance in both age groups. The impairment of NVC is accompanied by an increase in functional connections during the highest cognitive load, likely reflecting a compensatory mechanism. These results provide valuable insights into the physiological adaptations to cognitive challenges and the vascular mechanisms of neurocognitive aging.

Our findings have important implications for designing future clinical trials aimed at promoting healthy brain aging through cerebrovascular rejuvenation. The observed functional alterations can be non‐invasively assessed and potentially modifiable through interventions targeting NVC. As such, we have a compelling rationale for testing such interventions to preserve cognitive function in older adults. If successful, evaluating the effects of these interventions on neurovascular and cognitive endpoints in patients with MCI and VCI could be an important next step.

## 4 Conclusion

In this cross‐sectional study, we identify cerebrovascular alterations underlying age‐related cognitive dysfunction. In agreement with our preclinical studies, our data suggest that impaired neurovascular coupling responses underly impaired working memory performance in community‐dwelling older adults. These changes are accompanied by increased functional connectivity in the prefrontal cortex which can reflect a compensation of functional brain networks. Our results pave the way for clinical trials with interventions targeting cerebral hemodynamics and functional brain networks for preventing or delaying cognitive deterioration in high‐risk geriatric population.

## 5 Experimental Section

### Study Participants

Control young (*n* = 25, 21–45 years of age, 10 females, 15 males) and community‐dwelling older adults (*n* = 35, >65 years of age, 22 females, 13 males) were recruited in this cross‐sectional study. The study was conducted in the Translational Geroscience Laboratory of the Center for Geroscience and Healthy Brain Aging at the University of Oklahoma Health Sciences Center. The cognitive status and medical history of the candidate participants were screened to determine eligibility prior to participation. Inclusion criteria consisted of the ability to read and write in English, adequate hearing and visual acuity necessary for the examinations and competence to provide informed consent. Individuals with any of the following conditions were excluded: dementia (Mini Mental State Examination Score < 21), uncontrolled hypertension, uncontrolled diabetes mellitus, history of cerebrovascular or significant cardiac disease (e.g., heart failure), multiple sclerosis, chronic obstructive pulmonary disease, active cancer, established vascular disease, clinically significant anemia (<10 g dL^−1^). Participants refrained from consuming caffeinated beverages or any substances affecting the alertness for at least 6 hours prior to physiological assessments. All participants were enrolled in the study after obtaining signed informed consent. All procedures and protocols were approved by the Institutional Review Board of the University of Oklahoma Health Sciences Center (No. 9555).

### Measurement Protocol

**Figure 8:**
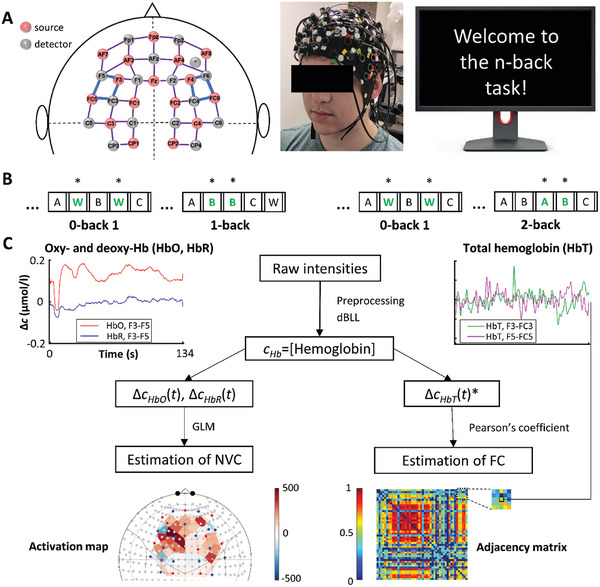
Schematic overview of measurement and analysis protocol for neurovascular coupling (NVC) and functional connectivity (FC) assessments. All results are based on data from 21 young and 30 aged participants. A) Cerebral hemodynamics were recorded from the frontal brain cortex using functional near‐infrared spectroscopy (fNIRS). The montage on the left shows the position of 16 light sources (pink) and 16 detectors (green) that define 48 measurement channels with 3 cm source‐detector separation shown as purple edges; blue edges denote the dorsolateral prefrontal cortex (DLPFC). The photo in the middle shows the fNIRS cap on the head of the participant and was set up according to the montage. Before the test, participants were seated in front of a computer that displayed instructions and administered the test. B) The cognitive stimuli were presented as part of a visual *n*‐back paradigm consisting of 0‐back, 1‐back, 0‐back and 2‐back sessions targeting the working memory domain. In brief, participants were presented with four tasks in the following order: 0‐back, 1‐back, 0‐back, and 2‐back. During each *n*‐back session, participants were asked to identify and respond by clicking the left mouse button to the target letter (shown in green bold), which was a letter "W" for *n* = 0 or the letter matching the *n* ^th^ letter preceding it for *n* = 1 or *n* = 2. C) Data processing at the individual level. Noise and trend were filtered from raw near‐infrared intensities during preprocessing, and differential Beer‐Lambert Law yielded relative concentration signals of oxyhemoglobin (HbO), deoxyhemoglobin (HbR) and total hemoglobin (HbT = HbO+HbR). Representative recordings from a young participant during the 2‐back task are shown on the left (HbO, HbR) and on the right (HbT). NVC responses were assessed using a General Linear Model (GLM) approach; the corresponding brain activation is characterized by regression coefficients (β weights) of the model. For FC analysis, Pearson correlation coefficients were determined for each pair of HbT* signals from different channels yielding an adjacency matrix from which brain network properties were derived in subsequent steps. For further details of the analyses, see the main text.

The measurement paradigm and the analytical pipeline is illustrated in **Figure** **8**. To record cerebral hemodynamic signals in the brain cortex, a NIRScout platform (NIRx Medical Technologies LLC, NY, USA) was utilized similarly to the previous studies.^[^ ^32^, ^44^ ^]^ This is a continuous wave instrument^[^ ^45^ ^]^ that allows for non‐invasive monitoring of changes in the optical density of the probed brain cortical regions, mainly due to changes in oxy‐ and deoxyhemoglobin concentration (HbO and HbR, respectively). The applied montage consisted of 16 sources and 16 photodetectors that covered the frontal cortex according to the international 10–20 system (Figure 8A) as described in ref. [44] Each channel consists of a source and detector that are separated by 3 cm enabling a sufficient penetration depth—1.5 cm—in the brain cortex. A short separation channel was defined to estimate scalp hemodynamics between the AF8 and AFF6h positions. This arrangement of optodes defined 48 channels corresponding to brain regions in the PFC—including the dorsolateral prefrontal cortices (DLPFC)^[^ ^28^ ^]^—and in the medial motor cortices on both sides, as described earlier.^[^ ^32^ ^]^ The optodes were embedded in an elastic cap (Easycap, Easycap GmbH, Woerthsee‐Etterschlag, Germany) that was mounted over the head by aligning its Fpz‐Iz line and the Fpz port with the midsagittal plane and Fpz position of the participant, respectively. All fNIRS signals were recorded with a sampling frequency of 3.9 Hz in a darkened and quiet room.

Simultaneously, a cognitive paradigm targeting the working memory domain was administered. The protocol consisted of visual *n*‐back sessions of various difficulties (Figure 8B), which has been well‐established in cognitive neuroscience research. After the paradigm was explained in detail to participants, the *n*‐back sessions were presented by the ePrime 3 software (Psychology Software Tools, Sharpsburg, PA) in the following order: 0‐back, 1‐back, 0‐back, and 2‐back. During the *n*‐back task, participants had to continuously remember a series of rapidly flashing letters (60 letters/session, interleaved by a pause of 250 ms) that were displayed on the screen and presented by a custom script in a randomized sequence and for a random time interval (1950 ± 100 ms). Participants were requested to provide response only to target stimuli. The definition of target or non‐target stimulus depended on the actual session; generally, the *n*‐back task requires participants to react when a stimulus is a target, the same as the *n*‐th letter before the current stimulus letter (25% of the presented letters). In case of 0‐back, the target stimulus was “W”.

### Working Memory Function

Cognitive outcome measures for the *n*‐back paradigm were obtained for each session separately based on the responses provided by the participants. To characterize processing speed, the average reaction time (RT) was defined, defined as the duration between the appearance of the target letter and the response (left mouse button click, if it occurred). To measure performance the number of correct responses to target stimuli was divided by the total number of target stimuli yielding the hit rate (_HIT_), while the false‐alarm rate (_FA_) is defined as the number of incorrect responses divided by the number of nontarget stimuli. In this study, the accuracy index (*d’*) was only used, which combines these scores as: *d*′ = *z*_Hit_ − *z_FA_*, where *z* denotes z‐transform.^[^ ^46^ ^]^

Cognitive function was also evaluated using the Cambridge Neuropsychological Test Automated Battery (CANTAB), a comprehensive battery of standardized tests. The battery of tests is optimized to detect age‐related changes in fluid cognitive abilities including reaction time, practice, processing and psychomotor speed, sustained attention, executive function and various forms of memory (visual episodic, verbal recognition, short‐term, working), see Supporting Information.

### Preprocessing of fNIRS Recordings

In principle, a preprocessing pipeline that enhances the neural activity‐related hemodynamic component was adopted. The fNIRS data processing was carried out using custom scripts written by the authors in Matlab 2021b (Mathworks, Natick, MA, USA). First, quality control of the data was performed; channels were excluded if the coefficient of variation calculated from the raw intensity signal was more than 7.5% as recommended^[^ ^47^ ^]^ which is considered as unphysiological noise.^[^ ^48^ ^]^ Participants were excluded if any of the channels covering the DLPFC or more than 80% of channels met this criterion indicating poor contact between the optodes^[^ ^49^ ^]^ and the scalp similarly to our previous fNIRS study.^[^ ^44^ ^]^ Prior to analysis, a series of necessary preprocessing steps were applied (Figure 8C) to eliminate motion artifacts and other physiological signal components captured by fNIRS that do not originate from functional or spontaneous brain activation. From adjacent vascular regions, scalp hemodynamics contribute to the measured signal that could be specifically captured by source–detector pairs with short separation; such signals were subtracted from the remaining channels probing the brain cortex. Moreover, systemic components contaminate all channels uniformly and can be attributed to physiological processes due to the function of the autonomic nervous system, including cardiac and respiratory activity. Their oscillatory nature separates them from low‐frequency fluctuations and hemodynamic responses due to neuronal activity; thus, these components of non‐neuronal origin can be effectively eliminated via band‐pass filtering. Subsequently, pre‐processed optical densities were converted to changes in chromophore concentrations using a modified version of the Beer‐Lambert law,^[^ ^50^ ^]^ which was also adjusted for age according to Equation 7 in ref. [51] Finally, evoked hemodynamic responses feature anticorrelated changes in hemoglobin compartments that can be distinguished from effects of non‐neuronal origin, as HbO and HbR typically change in the same direction in these cases.

### Analysis of NVC Responses

Specific steps of NIRS signal preprocessing were implemented in a pipeline created using the Brain AnalyzIR toolbox (commit 46c645d).^[^ ^52^ ^]^ The optical densities of the probed brain regions were transformed into time series of HbO and HbR, which were pre‐whitened using an autoregressive model‐based algorithm to attenuate serially correlated effects originating from systemic physiology and motion artefacts as described.^[^ ^52^, ^53^ ^]^ Slow drifts were eliminated using a discrete cosine transform‐based high‐pass filter (0.0045 Hz).

NVC responses were analyzed using a pipeline based on General Linear Model (GLM) approach, as described.^[^ ^32^ ^]^ To assess the dynamics of NVC responses, we set the stimulus duration to the whole length of each session (≈134 s) and used shorter stimulus windows with durations of 10, 30, 60 and 90 s beginning from the onset of the corresponding *n*‐back task. Brain activation is modeled as the convolution of the design matrix—describing the *n*‐back paradigm and preprocessing steps—with a canonical hemodynamic response. By fitting a GLM to HbO and HbR signals at the individual (1^st^) level, estimates of subject‐specific regression coefficients (β) were obtained for each *n*‐back session, chromophore and brain region.

### Characterizing Functional Brain Networks

In case of FC analysis, the short‐channel corrected intensity signals were first filtered after discrete wavelet transformation^[^ ^44^, ^54^ ^]^ and then in the frequency domain using a fifth‐order Butterworth filter with a band‐pass frequency range of 0.0045‐0.4 Hz. These steps reduced the contribution of motion artefacts and other systemic physiological processes to the measured hemodynamic fluctuations. HbO and HbR signals were then obtained using the modified Beer‐Lambert law,^[^ ^50^ ^]^ and their sum yielded a total hemoglobin (HbT) signal. To enhance the representation of signal components reflecting functional brain activity, a correlation‐based signal improvement was applied that eliminated correlated HbO‐HbR fluctuations typically due to motion.^[^ ^36^ ^]^ This final preprocessing step yielded a composite hemoglobin signal as the sum of neural activity‐related HbO and neural‐activity‐related HbR signal, as described.^[^ ^55^ ^]^

Correlations between such neural‐activity related HbT time series were quantified for the whole duration of each *n*‐back session (≈134 s) in terms of pairwise Pearson‐coefficients (*r*). for all channel pairs as described in ref. [55]. This step produced an adjacency matrix representing the connectome comprised of all connections between the corresponding brain regions. Next, spurious connections were eliminated by assigning 0 to negative *r* values; this thresholding approach assumes that only positive Pearson‐coefficients with *p* < 0.05 (not corrected for multiple comparisons) represent genuine functional interaction between the concerned brain regions.^[^ ^55^ ^]^ Both weighted and binarized adjacency matrices were used in the subsequent calculations implemented in the Brain Connectivity Toolbox,^[^ ^56^ ^]^ where connections passing the thresholding step—i.e., with non‐zero values—were assigned the unchanged Pearson‐coefficient or 1, respectively.

Here graph theoretical analysis was used to characterize the topology of functional brain networks, which regards each measurement channel as a node and each functional connection as an edge (link). Binary and weighted graph theoretical parameters that reveal the number and strength of connections, respectively, were calculated. These network metrics reflect local and global connectedness, for further details the reader is referred to refs. [56, 57]. Mathematically, each adjacency matrix defines a graph which is a snapshot of the connectome for the given participant and *n*‐back session. Considering the relatively small size of nodes within such a “brain graph,” only local and global network metrics characterizing the connectedness were obtained. All reported graph theoretical parameters are normalized to the maximum possible strength or number of connections of a given node for weighted and binary cases, respectively and thus take a value between 0 and 1. According to Equation (1), the normalized connection strengths for each node (*^w^D_i_*) and their average for the whole graph ($w\bar{D}$ ) were calculated, which are local and global network metrics, respectively.

(1a) $$wD_{i}=\frac{1}{N_{\mathrm{Ch}}}\sum_{j\;=\;1}^{N_{\mathrm{Ch}}}r_{ij}$$

(1b) $$w\bar{D}=\frac{1}{N_{\mathrm{Ch}}-1}\sum_{i}wD_{i}$$

Here *N*_Ch_ denotes the number of channels (*N*_Ch_ = 48), which is used for the normalization step; superscript *^w^* indicates these are weighted network metrics, while *i* and *j* are indices referring to a given node. Similarly, normalized local node degree (*D_i_*) and normalized global node degree ($\bar{D}$) were obtained by substituting values from a binarized adjacency matrix that consists of surrogate thresholded (*) Pearson‐correlation coefficients, $r_{ij}^{*}$.

(2a) $$\;D_{i}=\frac{1}{N_{\mathrm{Ch}}}\sum_{j\;=\;1}^{N_{\mathrm{Ch}}}r_{ij}^{*}$$

(2b) $$\;\bar{D}=\frac{1}{N_{\mathrm{Ch}}-1}\sum_{i}\;D_{i}$$

In the next step, the dynamics of the brain network at different rates was investigated. Since cognitive processes induce the reorganization of functional connections, the underlying brain network topology of neural coupling varies with time. Hence, the obtained graph theoretical parameters—as described in Equations 1 and 2—depend on the duration of the time window used for the estimation of FC. To reveal the temporal variation of $w\bar{D}$, and $\;\bar{D}$; a sliding window approach was applied for simultaneous neural‐activity‐related hemoglobin signals of various durations as described.^[^ ^58^ ^]^ The sliding window analyses were carried out with an overlap between subsequent analytical windows as the sliding step was one data point. Such dynamic analysis of FC ensured the highest temporal resolution and was performed for 10 s, 30 s, 60 s and 90 s time windows. Finally, mean and variance were computed as statistics characterizing the dynamic functional connectivity (DFC).

### Statistical Analyses

For group‐level analysis of NVC responses, the Brain AnalyzIR toolbox was used. All other statistical tests were performed with TIBCO Statistica 14.0 (TIBCO Software Inc., Palo Alto, California, USA). These analyses aimed to reveal the impact of aging on all measured and calculated outcomes and assess the relationship between parameters obtained from cognitive testing and fNIRS measurements. All analyses were repeated after adjusting to the effect of sex and education, by including either of these as an additional categorical term in the model at a group level.

In case of NVC responses, individual β weights—obtained as outputs of the 1^st^ level GLM—were compared between age groups (independent variable) and across n‐back sessions. Then a mixed effect model (2^nd^ level GLM) was fitted according to the Wilkinson‐Rogers formula of ‘β ∼ ‐1 + Group:session + (1|Subject)’ yielding group‐level statistics. This output was evaluated for each channel by defining t‐contrasts: i) to compare the young and elderly groups with an unpaired two‐tailed t‐test, and ii) to compare *n*‐back sessions repeated at two different difficulty levels with paired *t*‐tests. To avoid the type I error due to multiple (channel‐wise) comparisons, NVC responses were considered significant at *q* < 0.05 after controlling the false discovery rate (FDR) by Benjamini‐Hochberg‐procedure. To this end, the employed pipeline is able to reveal age‐group related differences in NVC responses by simultaneously evaluating the within‐subject effect of the task in which neurovascular coupling is in fact the same.

In all other cases, parametric statistical tests were used only if the Shapiro‐Wilk test confirmed the normal distribution. The unpaired two‐tailed *t‐*test was used to assess the impact of aging on cognitive outcome measures and functional brain network metrics, and Welch‐correction was applied in case of inhomogeneous variances checked by the Levene‐test. For reaction time, accuracy, $w\bar{D}$, $\;\bar{D}$: a GLM‐based approach was employed to assess the difference between the independent age groups and across dependent *n*‐back sessions with repeated measures. The Greenhouse‐Geisser correction was applied in case of sphericity violation checked by the Mauchley‐test. Tukey and Newman‐Keuls post hoc tests were used for the assessment of group (age) and task (*n*‐back session) effects. The nonparametric alternative for the unpaired *t*‐test was the two‐tailed Mann‐Whitney test comparing independent age groups, while dependent samples from different *n*‐back sessions were compared by a separately performed non‐parametric Friedman‐test.

## Conflict of Interest

The authors declare no conflict of interest.

## Supporting information

Supporting Information

## Data Availability

The data that support the findings of this study are available from the corresponding author upon reasonable request.
