# Supplementary material for: Impaired Neurovascular Coupling and Increased Functional Connectivity in the Frontal Cortex Predict Age‐Related Cognitive Dysfunction
Source: Adv Sci (Weinh). 2023 Dec 28;11(10):2303516. doi: 10.1002/advs.202303516 (PMC10962492; doi:10.1002/advs.202303516)
Supplement: Supplementary file 1 — Supporting Information [file ADVS-11-2303516-s001.pdf]

ADVANCED SCIENCE
Open Access

Supporting Information

for *Adv. Sci.*, DOI 10.1002/advs.202303516

Impaired Neurovascular Coupling and Increased Functional Connectivity in the Frontal Cortex Predict Age-Related Cognitive Dysfunction

*Peter Mukli, Camila B. Pinto, Cameron D. Owens, Tamas Csipo, Agnes Lipecz, Zsofia Szarvas, Anna Peterfi, Ana Clara da Costa Pinaffi Langley, Jordan Hoffmeister, Frigyes Samuel Racz, Jonathan W. Perry, Stefano Tarantini, Ádám Nyúl-Tóth, Farzaneh A. Sorond, Yuan Yang, Judith A. James, Angelia C. Kirkpatrick, Calin I. Prodan, Peter Toth, Juliette Galindo, Andrew W. Gardner, William E. Sonntag, Anna Csiszar, Zoltan Ungvari and Andriy Yabluchanskiy**

Supporting Information

Impaired neurovascular coupling and increased functional connectivity in the frontal cortex predict age-related cognitive dysfunction

Peter Mukli[1,2,3,4,#], Camila B. Pinto[1,2,#], Cameron Owens[1,2,#], Tamas Csipo[1,2,4,5,#], Agnes Lipecz[1,2,4,#], Zsofia Szarvas[1,2,4], Anna Peterfi[1,2,4], Ana Clara da Costa Pinaffi Langley[1,2], Jordan Hoffmeister[5], Frigyes Samuel Racz[3,6], Jonathan W. Perry[1], Stefano Tarantini[1,2,4,7,8], Ádám Nyúl-Tóth[1,2,4], Farzaneh A. Sorond[9], Yuan Yang[10,11,12], Judith A. James[1,13,14,15], Angelia C. Kirkpatrick[16], Calin I. Prodan[16,17], Peter Toth[4,18,19,20], Juliette Galindo[5], Andrew W. Gardner[21], William E. Sonntag[1], Anna Csiszar[1,2,4,8], Zoltan Ungvari[1,2,4,7,8], Andriy Yabluchanskiy[1,2,7,8,]*

Standardized cognitive assessments

All study participants were administered a selection of tests from the Cambridge Neuropsychological Test Automated Battery (CANTAB, Cambridge Cognition) at the beginning of the visit, as previously described.[S1] The CANTAB Connect Research tool is validated to reliably identify the earliest signs of age-related changes in cognitive performance.[S2] The battery of tests is optimized to detect age-related changes in cognitive domains mainly concerning fluid abilities, including reaction time, practice, attention, memory (visual episodic and verbal recognition memory, attention, short-term memory, visual recognition), and executive function (working memory and strategy). Each assessment began with a motor screening test (MOT) and continued with the following tests from the battery: Delayed Match to Sample (DMS, short-term visual memory), Paired Association Learning (PAL, visual memory, and learning), Reaction Time (RTI, psychomotor speed), Rapid Visual Processing and Attention (RVPA, sustained attention), Spatial Working Memory (SWM, working memory and strategy). Below a brief description of these tests is provided. After evaluating sensorimotor function and comprehension with MOT, the participant was administered the DMS test and instructed to match the target object with visually identical stimuli presented together with visually similar items, simultaneously or with delay (0, 4, 12 seconds). In the case of the PAL test, the participant was required to recall the location previously paired with an object. RTI and RVPA tests measured the response to a target stimulus, which is an object at a specific location presented simultaneously with non-target objects or a given sequence of successively presented numbers in a series of numbers with non-matching sequences, respectively. Finally, the SWM test implemented in CANTAB specifically assessed the participant's ability to find all hidden tokens in each number of boxes in the lowest number of attempts opening attempt.

Table S1. *Age group-specific descriptive statistics and comparison of all cognitive outcome measures obtained by CANTAB performance between young and elderly participants.* Unpaired comparisons revealed significant differences in all assessed cognitive domains.

	**Young (*n*=21)**		**Elderly (*n*=30)**		**Test name**	**Statistics* (*df*)**	***p*-value**	**Effect size**
	**Mean ±SD**	**Median [IQR]**	**Mean ±SD**	**Median [IQR]**				
DMSPC	93.10 ±5.356	95.00 [5.000]	82.17 ±8.678	82.50 [15.00]	MW	96.0	<0.0001	0.6952
DMSMDL	2720 ±577	2839 [862]	3653 ±1091	3609 [1300]	*t*-test (S)	3.576 (49)	0.0008	1.0176

PALFAMS28	15.714 ±3.196	15.00 [4.000]	8.567 ±3.569	9.500 [6.000]	*t*-test (S)	-7.342 (49)	<0.0001	-2.089
PALTEA28	8.400 ±10.26	5.500 [7.00]	28.93 ±14.05	22.50 [27.00]	MW	45.0	<0.0001	0.8500
RTIFMDMT	258.2 ±51.95	253.5 [61.50]	344.6 ±101.3	319.5 [167.5]	*t*-test (W)	3.980 (45.5)	0.0008	1.0726
RTIFMDRT	353.2 ±32.45	349.5 [35.50]	373.4 ±50.07	368.5 [53.00]	*t*-test (S)	1.622 (49.0)	0.1111	0.4616
RVPA	0.933 ±0.039	0.926 [0.046]	0.847 ±0.060	0.851 [0.077]	*t*-test (S)	-5.719 (49)	<0.0001	-1.6387
RVPMDL	403.6 ±37.57	406.5 [41.00]	560.4 ±142.0	546.0 [159.0]	*t*-test (W)	5.679 (33.2)	<0.0001	1.5099
SWMBE468	3.850 ±7.013	0.000 [5.500]	19.37 ±8.240	19.00 [11.00]	MW	57.0	<0.0001	0.8100
SWMS	5.429 ±2.856	6.000 [6.000]	9.133 ±1.961	9.000 [2.000]	MW	89.0	<0.0001	0.7175

Bold denotes significant differences for the corresponding variable name ($p<0.05$, two-sided test). S: Student's t-test, W: Welch's t-test, MW: Mann-Whitney U test. DMS: Delayed Match to Sample, PC: percent correct for all seconds delay and in case of simultaneous presentation of target and stimuli; PAL: Paired Associates Learning, FAMS: first attempt memory score (*PALFAMS28*), TEA28: adjusted number of total errors calculated across all assessed trials (*PALTEA28*); RTI: Reaction Time, FMDRT: Median Five-Choice Movement Time (*RTIFMDRT*), FMDMT: Median Five-Choice Movement Time (*RTIFMDMT*); RVPA: Rapid Visual Processing and Attention, MDL: median response latency (*RVPMDL*), SWM: Spatial Working Memory, BE: between errors calculated as the number of an unnecessary revisit of the previously selected box across all assessed 4, 6 and 8 token trials (*SWMBE468*), *SWMS*: SWM strategy, the number of times a subject begins a new search pattern from the same box they started with previously. If they always begin a search from the same starting point, we infer that the subject employs a planned strategy for finding the tokens. Therefore, a low score indicates high strategy use (1 = they always begin the search from the same box), and a high score indicates that they are beginning their searches from many different boxes. Calculated across assessed trials with 6 tokens or more.

Table S1 reports the key outcome measures for the assessed cognitive domains reflecting fluid abilities in the study population. All participants successfully completed the screening tests, which indicated no significant age-related difference in the sensorimotor function of young and elderly persons. We found a statistically significant age effect in the performance of all neuropsychological tests (DMS, PAL, RTI, RVP, SWM) assessing different cognitive domains. The percentage of correct responses during the Delayed Matching Sample test (*DMSPC*) was lower in the elderly group indicating poorer memory function (non-verbal). We also observed a significantly worse performance on a PAL test in the elderly group (*PALFAMS* – higher value means better performance, *PALTEA28* – higher value means worse performance), which also implies memory impairment (specifically, episodic memory). *RTIFMDMT* and *RVPMDL* are key measures of processing speed that were significantly lower in the elderly group. RVP test also demonstrated attention deficits and slower processing in the aged group, given their lower RVPA and higher RVPPFA scores. Finally, we observed more errors during the Spatial Working Memory paradigm in older adults, captured by *SWMBE468* (BE468 is a composite score referring to a number of errors for all mandatorily administered task conditions) parameters along with lower strategy score (*SWMS*), indicating poor working memory and executive function. CANTAB Connect Tool provides additional standardized measures that allow for a comprehensive characterization of different cognitive domains, further revealing the impact of aging on cognition. The readers are encouraged to download the data files characterizing cognitive outcome measures obtained by CANTAB Connect Research

Tool from Physionet [project title: “Functional near infrared spectroscopy data recorded during n-back memory task and cognitive outcome measures from healthy young and older adults”].

Relationship between cognitive outcome measures, age and fNIRS parameters

In contrast to the *n*-back paradigm administered during fNIRS measurements, CANTAB Connect Research Tool provides metrics for different domains of cognitive function that are standardized. The relationship of these metrics is of interest for evaluating the domain-specific impact of aging on cognition. Therefore, we correlated RT and *d’* with key cognitive outcome measures yielded by the CANTAB Connect Research Tool (**Table S2**). We also found a significant association between *d’* corresponding to 2-back condition and key performance measures (DMSPC, PALFAMS28, PALTEA28, RVPA, SWMBE468, SWMS) in all assessed cognitive domains; in case of error score, these were inversely related. Measures of reaction latency (DMSMDL, RVPMDL) and reaction time, including the motor component of the response (RTIFMDMT), were also significantly correlated with 2-back RT. Please note that the correlation within the whole population is typically stronger than within age groups. On the one hand, we assume that the lower age-specific correlations can be attributed to the different difficulties of the tasks: either too easy for young participants or too hard for the elderly. On the other hand, the higher contribution of age-independent (assessment-specific) factors to the variability of these outcome measures may account for less correlated cognitive outcome measures, as in this case, age is not driving the relationship. The inference is that both tasks capture the age-related impairment of the cognitive performance, and while the *n*-back paradigm is assumed to measure working memory function, it correlates with other measures of fluid cognitive abilities typically declining with aging.

Table S2. *Relationship between cognitive outcome measures yielded by CANTAB Connect Research Tool and verbal n-back paradigm for the young group, elderly group and for the whole study population.* Correlation analyses revealed significant associations in all assessed cognitive domains.

**Cognitive outcome measures**			**Rank correlation analysis**	
**CANTAB**	***n*-back paradigm**	**Group**	**Strength (r*ho*)**	**Probability level (*p*)**
DMSMDL	*RT* 2-back	*Y*	0.4338	0.0508
		E	0.5220	0.0031
		Y+E	0.5532	<0.0001
RTIFMDMT		*Y*	-0.0364	0.8755
		E	0.3024	0.1044
		Y+E	0.3217	0.0213
RTIFMDRT		*Y*	0.1792	0.4371
		E	0.1799	0.3414
		Y+E	0.1894	0.1831
RVPMDL		*Y*	0.2481	0.2783
		E	0.3269	0.0835
		Y+E	0.4568	0.0009
DMSPC	*d'* 2-back	*Y*	0.2584	0.2581
		E	0.0905	0.6342
		Y+E	0.5172	0.0001
PALFAMS28		*Y*	0.4018	0.0710
		E	0.6219	0.0002
		Y+E	0.7771	<0.0001
PALTEA28		*Y*	-0.3799	0.0985
		E	-0.5249	0.0029
		Y+E	-0.8295	<0.0001

	Y	0.3202	0.1570
RVPA	*E*	0.4409	0.0167
	Y+E	0.6980	<0.0001
	Y	-0.220	0.3513
SWMBE468	*E*	-0.3095	0.0961
	Y+E	-0.6361	<0.0001
	Y	0.0540	0.8162
SWMS	*E*	-0.2345	0.2124
	Y+E	-0.5244	<0.0001

Bold denotes significant differences for the corresponding variable name (p<0.05 of Spearman's rho). *Y*: young, *E*: elderly group, *Y+E* refers to all study participants. DMS: Delayed Match to Sample, PC: percent correct for all seconds delay and in case of simultaneous presentation of target and stimuli; PAL: Paired Associates Learning, FAMS: first attempt memory score (*PALFAMS28*), TEA28: adjusted number of total errors calculated across all assessed trials (*PALTEA28*); RTI: Reaction Time, FMDRT: Median Five-Choice Movement Time (*RTIFMDRT*), FMDMT: Median Five-Choice Movement Time (*RTIFMDMT*); RVPA: Rapid Visual Processing and Attention, MDL: median response latency (*RVPMDL*), SWM: Spatial Working Memory, BE: between errors calculated as the number of an unnecessary revisit of the previously selected box across all assessed 4, 6 and 8 token trials (*SWMBE468*), *SWMS*: SWM strategy, the number of times a subject begins a new search pattern from the same box they started with previously. If they always begin a search from the same starting point we infer that the subject is employing a planned strategy for finding the tokens. Therefore, a low score indicates high strategy use (1 = they always begin the search from the same box), and a high score indicates that they are beginning their searches from many different boxes. Calculated across assessed trials with 6 tokens or more.

To understand age-independent and age-specific associations, we utilized cognitive outcome measures from the *n*-back paradigm only from the 2-back task condition sensitive to group effect and performed correlation analysis with fNIRS parameters (**Table S3**). In the whole study population, only one parameter showed a significant association with β_{HbO} characterizing NVC response during the 2-back task: first attempt memory score, a key measure of the PAL test**.** Conversely, increased global connection strength associate with lower PALFAMS28 and higher error scores (PALTEA28). In case of $\overline{D}$, we found a significant negative correlation with PALFAMS28 and a positive correlation with PALTEA28, performance and error scores of the PAL test, respectively. Age-group specific analyses confirm the positive correlation between PALFAMS28 and HbO responses during the 2-back task condition, which was the only significant relationship when data of young and aged participants were processed separately**.** Taken together, measures of impaired NVC responses and increased FC significantly associate with key variables of cognitive function yielded by the PAL test, while other performance metrics do not correlate with such fNIRS-based parameters.

Table S3. *Relationship between cognitive outcome measures yielded by CANTAB Connect Research Tool and fNIRS parameters obtained during 2-back task condition.* Correlation analyses revealed significant associations in the elderly group and in the whole study population for key measures of the Paired Associates Learning test.

	**fNIRS parameter**	**β_{HbO} 2-back**		**$\overline{D}$ 2-back**	
**CANTAB parameter**		**Correlation (*r* or *rho*)**	**Probability level (*p*)**	**Correlation (*r* or *rho*)**	**Probability level (*p*)**

Young	*DMSPC*	0.2887	0.2044	-0.2140	0.3516
	DMSMDL	0.0626 *P*	0.7875	0.3816*P*	0.0878
	PALFAMS28	0.1813*P*	0.4316	-0.2455*P*	0.2835
	PALTEA28	0.2662	0.2567	0.3191	0.1703
	RTIFMDMT	0.0019*P*	0.9936	-0.0560*P*	0.8097
	RTIFMDRT	0.0616	0.7907	-0.0934	0.6871
	RVPA	-0.2300*P*	0.3159	0.3242*P*	0.1516
	RVPMDL	-0.0281*P*	0.9039	-0.1337*P*	0.5635
	SWMBE468	0.2605	0.2674	-0.3717	0.1066
	SWMS	0.3096*P*	0.1721	-0.4267*P*	0.0537
Elderly	*DMSPC*	-0.2095*P*	0.2666	0.1089*P*	0.5668
	DMSMDL	0.1115*P*	0.5575	-0.0571*P*	0.7646
	PALFAMS28	**0.4702*P***	**0.0087**	-0.0862*P*	0.6506
	PALTEA28	-0.2631	0.1600	0.0945	0.6193
	RTIFMDMT	0.0993*P*	0.6081	0.1871*P*	0.3310
	RTIFMDRT	-0.1678	0.3753	0.0331	0.8619
	RVPA	0.0852	0.6532	-0.1132	0.5498
	RVPMDL	0.2832	0.1293	0.0919	0.6291
	SWMBE468	-0.3379*P*	0.0678	0.2026*P*	0.2829
	SWMS	-0.2171	0.24916	0.0844	0.6572
Young + Elderly	*DMSPC*	-0.046	0.750	-0.174	0.222
	DMSMDL	0.046*P*	0.746*P*	0.158*P*	0.268*P*
	PALFAMS28	**0.321*p***	**0.0221*p***	**-0.280*p***	**0.047*p***
	PALTEA28	-0.033*P*	0.822*P*	**0.281*P***	**0.048*P***
	RTIFMDMT	0.113	0.436	-0.093	0.522
	RTIFMDRT	-0.118*P*	0.409*P*	0.141*P*	0.322*P*
	RVPA	-0.083	0.560	0.177	0.213
	RVPMDL	0.121	0.400	0.097	0.500
	SWMBE468	-0.092	0.523	0.173	0.230
	SWMS	-0.060	0.679	0.039	0.784

Bold denotes significant ($p<0.05$) correlation coefficients (Spearman's rho or Pearson's correlation coefficient, the latter is denoted by subscript *P*). DMS: Delayed Match to Sample, PC: percent correct for all seconds delay and in case of simultaneous presentation of target and stimuli; PAL: Paired Associates Learning, FAMS: first attempt memory score (*PALFAMS28*), TEA28: adjusted number of total errors calculated across all assessed trials (*PALTEA28*); RTI: Reaction Time, FMDRT: Median Five-Choice Movement Time (*RTIFMDRT*), FMDMT: Median Five-Choice Movement Time (*RTIFMDMT*); RVPA: Rapid Visual Processing and Attention, MDL: median response latency (*RVPMDL*), SWM: Spatial Working Memory, BE: between errors calculated as the number of an unnecessary revisit of the previously selected box across all assessed 4, 6 and 8 token trials (*SWMBE468*), *SWMS*: SWM strategy, the number of times a subject begins a new search pattern from the same box they started with previously.

If they always begin a search from the same starting point, we infer that the subject employs a planned strategy for finding the tokens. Therefore, a low score indicates high strategy use (1 = they always begin the search from the same box), and a high score indicates that they are beginning their searches from many different boxes. Calculated across assessed trials with 6 tokens or more.

Analysis of confounding variables

Sex-related differences in neurovascular coupling responses and functional connectivity

Neurovascular coupling (NVC)-related hemodynamic responses evoked by cognitive *n*-back task were recorded by functional near-infrared spectroscopy (fNIRS). Oxy- and deoxyhemoglobin (HbO and HbR, respectively) were compared between male and female participants. With the aid of an fNIRS analytical pipeline similar to what was used to assess the impact of aging, we obtained HbO and HbR maps of *t*-statistics for the male-female contrast. Analyses were carried out separately for the young and elderly groups. The impact of sex was significant in three circumscribed, separate frontal cortical areas suggesting increased NVC responses in young females compared to young males and decreased NVC responses in elderly females compared to elderly males (**Figure S1**). Detailed descriptive statistics are provided as **Supplementary Data File.**

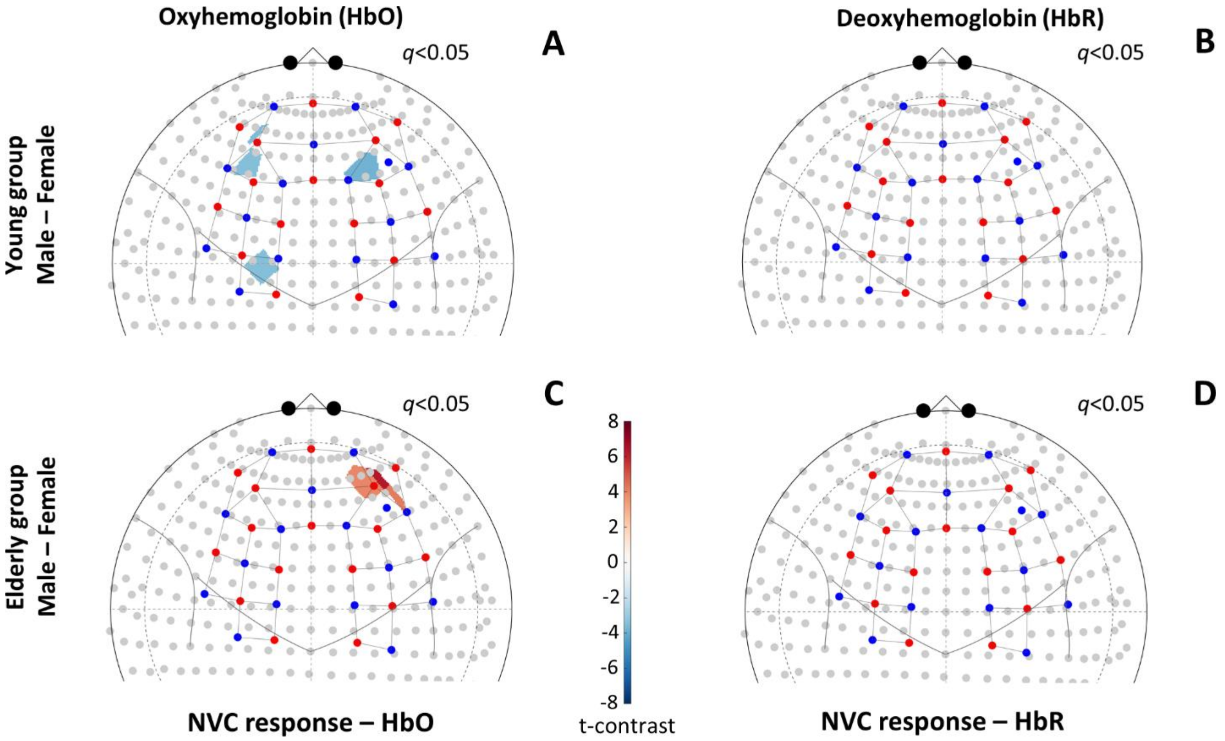

Figure S1. *Impact of sex on neurovascular coupling (NVC) responses elicited by cognitive n-back task in the prefrontal and motor brain cortices.* Color-coded t-values are mapped with a cutoff at $q<0.05$ (obtained after false discovery rate correction) in the montage space. Significant sex-related differences revealed by the statistical contrast of HbO changes are localized both in the young (**A**) and in the elderly group (**C**). HbR maps do not show any differences between males and females, neither in the young group (**B**) nor in the elderly group (**D**).

Changes in global measures of functional connectivity (FC) during our cognitive n-back paradigm were compared between male and female participants. **Figure S2** shows the normalized global node degree and normalized connection strength characterizing the male and

female group for each session. Analyses were carried out separately for the young and elderly groups. Although GLM revealed a significant group effect ($F(1,49)=4.592$, $p=0.0372$), sex did not have a significant influence on any of these parameters, neither in the young nor in the aged group.

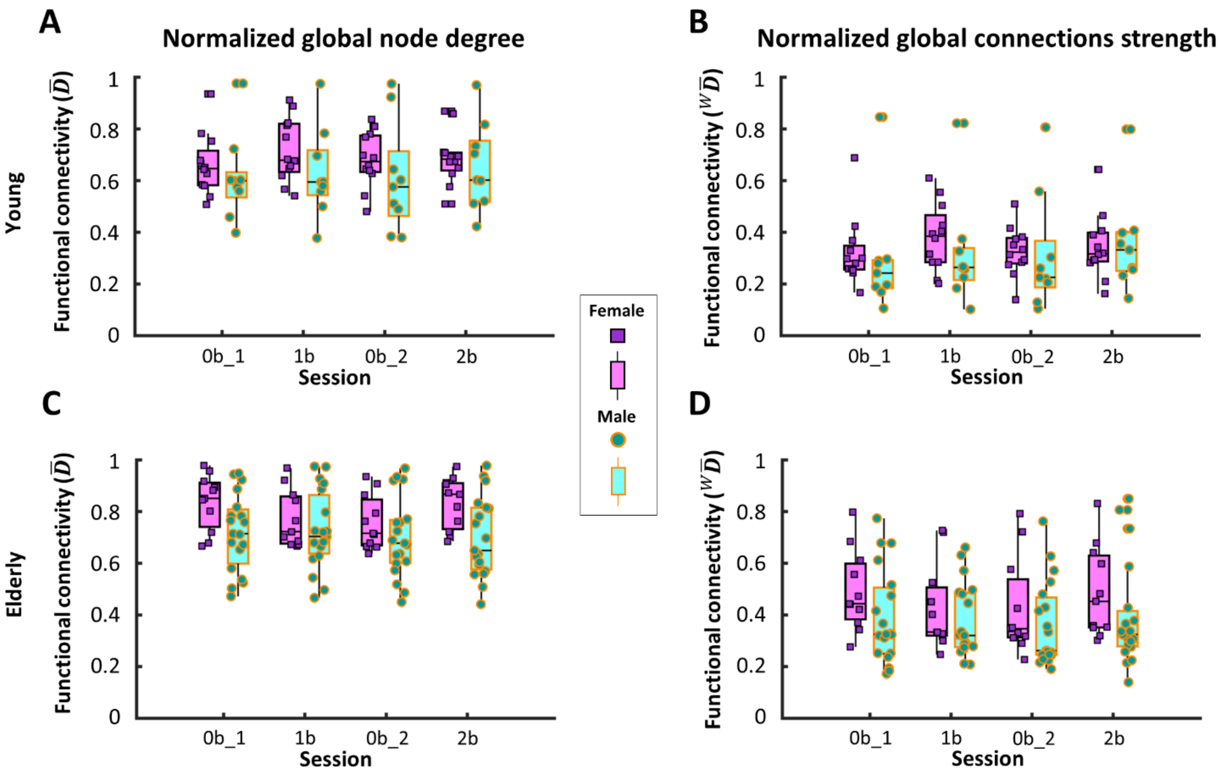

Figure S2. *Sex-related differences in global network metrics characterizing static functional connectivity (FC) in the frontal cortex during sessions of n-back task.* Individual global (i.e., referring to the whole frontal cortex) network metrics values are shown in dark purple squares (female group) or dark cyan dots (male group) separately for 0-back_1 (0b1), 1-back (1b), 0-back_2 (0b2) and 2-back (2b) sessions. Corresponding median values and interquartile ranges are displayed as magenta (female) or cyan (male) boxplots. **Panel A** and **Panel B** compare the normalized global node degree ($\overline{D}$) and normalized global connection strength ($^{W}\overline{\boldsymbol{D}}$) between young males ($n$=12) and young females ($n$=9), respectively. **Panel C** and **Panel D** compare $\overline{D}$ and $^{W}\overline{D}$ between elderly males (n=12) and elderly females (n=9), respectively. None of the sex-related differences were significant ($p>0.05$ for all comparisons); for further details, see supplementary text.

Effect of education on neurovascular coupling responses and functional connectivity

Since the level of education was considerably different between young and elderly groups, it may affect the primary outcome parameters obtained by fNIRS. To assess this effect we defined a group of participants with higher (having at least an MSc/MA degree) and lower level of education (everyone else). This categorization led to bins with sufficient number of observations: young participants with higher (*Hi*, n=13) or lower (*Lo*, n=8) level of education as well as n=7 and n=23 elderly participants in the *Hi* and *Lo* subgroup, respectively.

First, we checked whether higher education level was associated with better neurovascular coupling responses. NVC responses were analyzed with a pipeline similar to what we used to assess the effect of age or sex (**Figure S3**). We obtained HbO and HbR maps of t-statistics for

the *Hi-Lo* contrast. The impact of education level in the young group was significant in two focal areas in the prefrontal cortex suggesting altered NVC responses in participants with more education compared to young participants with less education. In the case of the elderly group, our analysis revealed frontal areas whose NVC responses were significantly higher in participants having higher level of education compared to those with lower level of education.

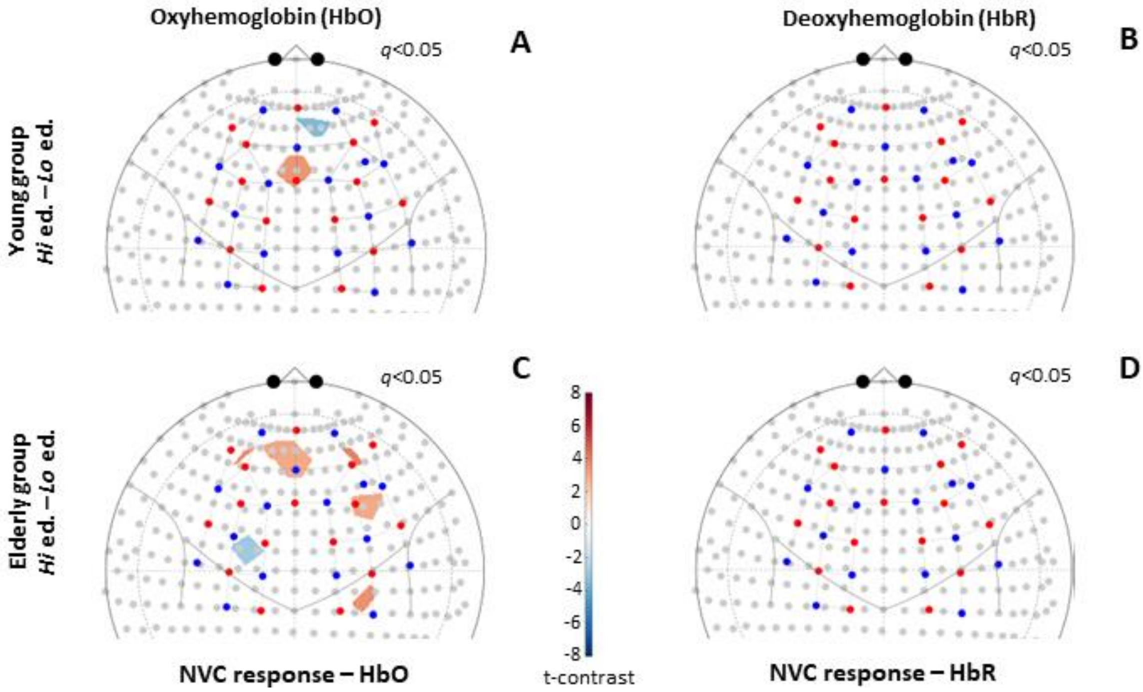

Figure S3. Impact of education on neurovascular coupling (NVC) responses elicited by cognitive n-back task in the prefrontal and motor brain cortices. *Hi* ed.: higher education level, refers to participants with who have at least an MSc/MA degree; *Lo* ed.: lower education level all, refers to everyone else. Color-coded t-values are mapped with a cutoff at $q<0.05$ (obtained after false discovery rate correction) in the montage space. Significant education level-related differences revealed by the statistical contrast of HbO changes are localized both in the young (**A**) and in the elderly group (**C**). HbR maps do not show any differences between males and females, neither in the young group (**B**), nor in the elderly group (**D**).

Changes in global measures of functional connectivity (FC) during our cognitive n-back paradigm were compared between participants with higher education level and participants with lower education level. **Figure S4** shows the normalized global node degree and normalized connection strength characterizing the *Hi* and *Lo* group for each session. Analyses were carried out separately for the young and elderly groups. Level of education did not have a significant impact on any global network metrics corresponding to different task conditions.

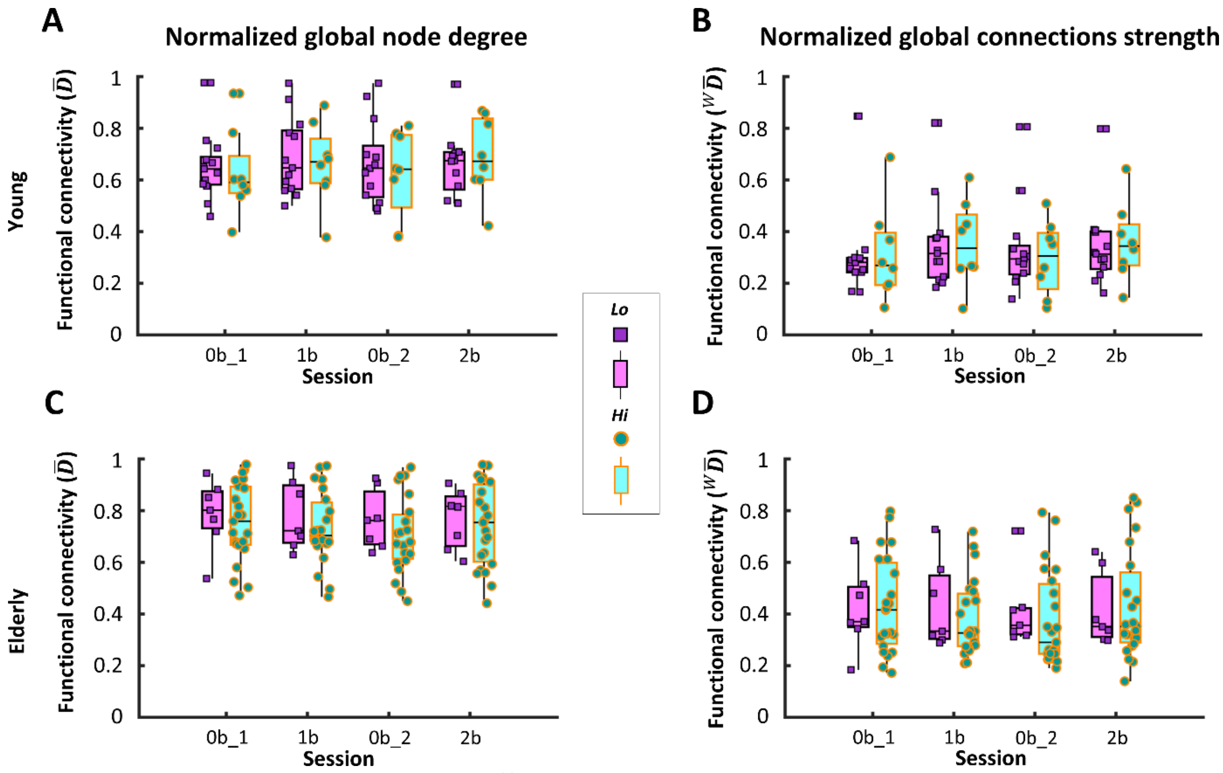

Figure S4. *Education level-related differences in global network metrics characterizing static functional connectivity (FC) in the frontal cortex during sessions of n-back task. Hi* ed.: higher education level, refers to participants with who have at least an MSc/MA degree; *Lo* ed.: lower education level all, refers to everyone else. Individual global (i.e., referring to the whole frontal cortex) network metrics values are shown in dark purple squares (*Lo* group) or dark cyan dots (*Hi* group) separately for 0-back_1 (0b1), 1-back (1b), 0-back_2 (0b2) and 2-back (2b) sessions. Corresponding median values and interquartile ranges are displayed as magenta (*Lo*) or cyan (*Hi*) boxplots. **Panel A** and **Panel B** compare the normalized global node degree ($\bar{D}$) and normalized global connection strength ($^W\bar{D}$) between young participants with more (n=13) and with less education (n=8), respectively. **Panel C** and **Panel D** compare $\bar{D}$ and $^W\bar{D}$ between elderly participants with higher education levels (n=7) and with lower education levels (n=23), respectively. None of the education level-related differences were significant (p>0.05 for all comparisons), for further details, see supplementary text.

Analysis of dynamic functional connectivity (DFC)

Age- and task-related changes in DFC in the frontal cortex

Dynamic analyses of FC revealed the impact of aging on the temporal evolution of functional brain networks reconstructed from correlated hemodynamics of different durations. **Figure S5** clearly demonstrates the effect of age group on $\overline{^WD_{loc}}(t)$, which is, however, similar across task conditions (p>0.05) and for different durations of the time window. Please observe that the obtained network metrics fluctuate in a higher range in the aged group during all n-back sessions (Figure S5A, S5C, S5E, S5G). Moreover, the connection strength is higher in the elderly group independently from the time window of interest along the $\overline{^WD_{loc}}(t)$.

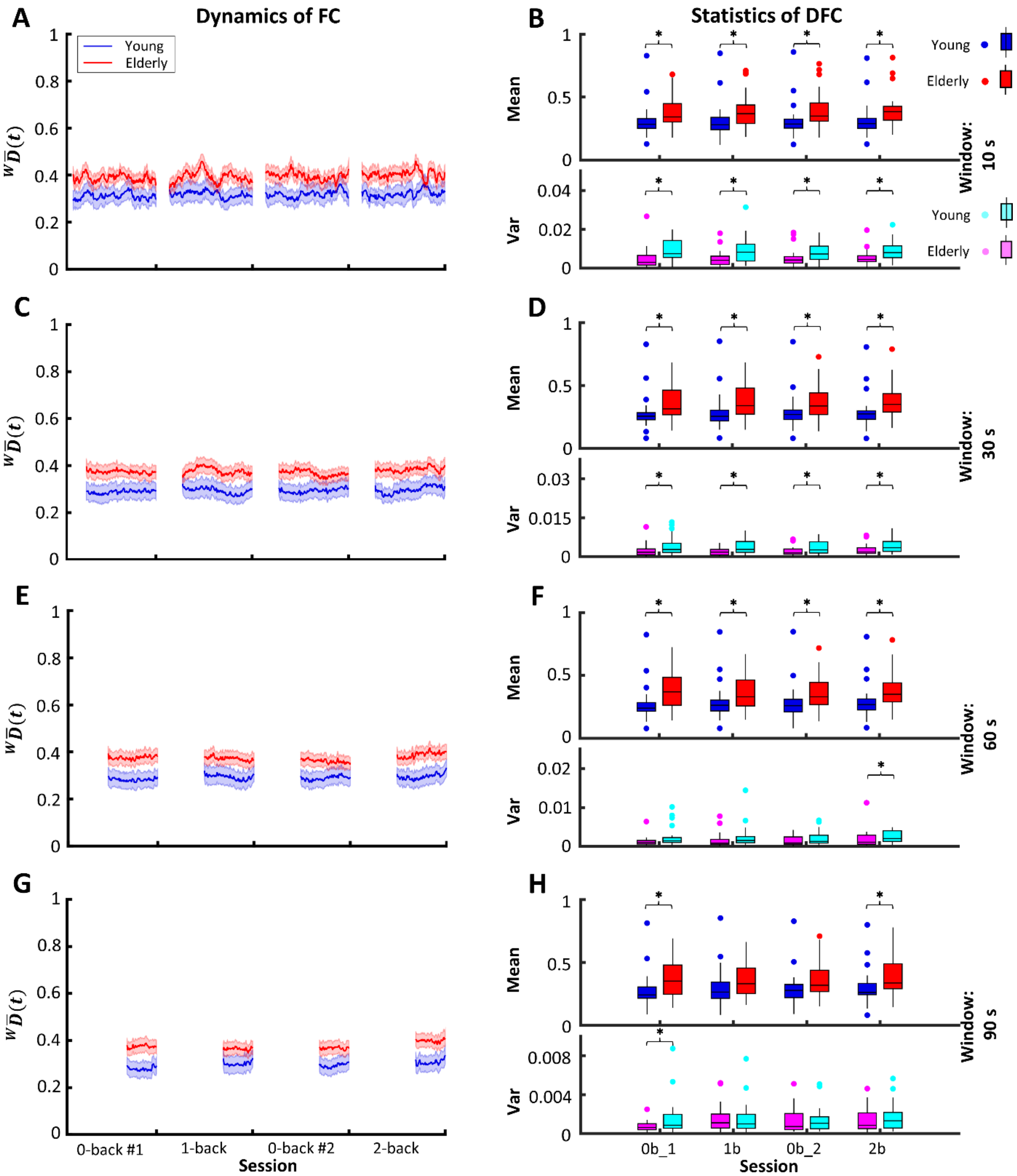

Figure S5. *Age-related changes in dynamic functional connectivity (DFC) in the frontal cortex during sessions of n-back task*. Dynamic functional connectivity (DFC) analysis was carried out in a sliding window manner – where each window has an ID referring to the actual functional brain network – yielding a series of ${}^{W}\overline{D}(t)$. The dynamics of ${}^{W}\overline{D}(t)$ is compared between the young (blue) and elderly groups (red); the thick line and shaded area refer to mean and standard error, respectively. Each panel shows the temporal pattern of network metrics for different time scales corresponding to the correlation window size of 10 (**A**), 30 (**B**), 60 (**C**) and 90 seconds (**D**). ${}^{W}\overline{D}(t)$ reveals a clear distinction between the trajectories of young and elderly participants during all sessions of the *n*-back paradigm captured in all window scales used for DFC analysis. The dynamics of normalized global network connection strengths (${}^{w}D_{norm}$) values are shown in blue squares (young group, *n*=21) or red dots (elderly group, *n*=30) separately for 0-back_1 (0b1), 1-back (1b), 0-back_2 (0b2) and 2-back (2b) sessions. Panels on the left depict the average of ${}^{W}\overline{D}(t)$ corresponding to correlation window size of 10 (**A**), 30 (**C**), 60 (**E**) and

90 seconds (**G**). Panels on the right depict the variance of $^{W}\overline{D}(t)$ corresponding to correlation window size of 10 (**B**), 30 (**D**), 60 (**F**) and 90 seconds (**H**). The impact of aging on global brain network dynamics was assessed by the Mann-Whitney U test; significant measures of dynamic FC are denoted by *, and *p*-values are reported in **Supplementary Table S4**. Notably, the young and elderly group differs for all *n*-back sessions in terms of mean and variance $^{W}\overline{D}(t)$ captured in 10 and 30 seconds, while task-related effects are not significant for any age group and any correlation window lengths.

Statistical tests were conducted on the mean and variance of $\overline{^{W}D_{loc}}(t)$, corresponding results are also reported in **Table S4**. Significant effect of age group was verified by the Mann-Whitney test on the mean of $\overline{^{W}D_{loc}}(t)$ for all task conditions analyzed at 10 (Figure S4B), 30 (Figure S4D) and 60 (Figure S4F) seconds, and for the first 0-back and 2-back conditions analyzed at 90 seconds (Figure S4H). Variance of $\overline{^{W}D_{loc}}(t)$ was significantly higher for all task conditions in the aged group corresponding to window sizes of 10 (Figure S4B) and 30 seconds (Figure S4D), for the 2-back condition at 60 seconds (Figure S4F) and for the first 0-back condition at 90 seconds (Figure S4H). In summary, the DFC of the aged persons is characterized by higher mean and increased variance at shorter time windows, which was disproportional at 10 sec in particular.

Table S4. *Descriptive statistics for dynamic functional connectivity analysis.* Trajectories of normalized weighted global node degree are characterized by their mean and variance for 10 sec, 30 sec, 60 sec and 90 sec time windows. According to the Mann-Whitney test, the age group effect is significant for all sessions in case of 10 sec and 30 sec time windows.

	**Window size (task)**	**Young (median [IQR])**	**Elderly (median [IQR])**	***p*-value**		**Window size (task)**	**Young (median [IQR])**	**Elderly (median [IQR])**	***p*-value**
**DFC – mean of $^{W}\overline{D}(t)$**	*10 s (0b1) **	0.284 [0.070]	0.342 [0.146]	0.002	**DFC – variance of $^{W}\overline{D}(t)$**	*10 s (0b1) **	0.003 [0.005]	0.007 [0.009]	0.005
	*10 s (1b) **	0.281 [0.090]	0.369 [0.147]	0.012		*10 s (1b) **	0.004 [0.004]	0.008 [0.009]	0.010
	*10 s (0b2) **	0.287 [0.068]	0.350 [0.145]	0.003		*10 s (0b2) **	0.004 [0.004]	0.007 [0.007]	0.006
	*10 s (2b) **	0.290 [0.076]	0.385 [0.110]	0.002		*10 s (2b) **	0.005 [0.003]	0.008 [0.006]	0.001
	*30 s (0b1) **	0.256 [0.053]	0.315 [0.195]	0.005		*30 s (0b1) **	0.002 [0.002]	0.003 [0.004]	0.032
	*30 s (1b) **	0.256 [0.073]	0.340 [0.207]	0.017		*30 s (1b) **	0.002 [0.002]	0.003 [0.005]	0.022
	*30 s (0b2) **	0.270 [0.070]	0.338 [0.173]	0.018		*30 s (0b2) **	0.002 [0.002]	0.003 [0.004]	0.034
	*30 s (2b) **	0.275 [0.062]	0.351 [0.146]	0.003		*30 s (2b) **	0.002 [0.002]	0.004 [0.004]	0.030
	*60 s (0b1) **	0.240 [0.059]	0.369 [0.221]	0.007		*60 s (0b1)*	0.001 [0.001]	0.001 [0.001]	0.087
	*60 s (1b) **	0.263 [0.079]	0.330 [0.206]	0.029		*60 s (1b)*	0.001 [0.001]	0.002 [0.002]	0.150
	*60 s (0b2) **	0.259 [0.093]	0.330 [0.176]	0.036		*60 s (0b2)*	0.001 [0.002]	0.001 [0.002]	0.327
	*60 s (2b) **	0.268 [0.082]	0.350 [0.151]	0.004		*60 s (2b) **	0.001 [0.002]	0.002 [0.003]	0.027
	*90 s (0b1) **	0.243 [0.080]	0.353 [0.232]	0.010		*90 s (0b1) **	0.001 [0.001]	0.001 [0.001]	0.032
	90 s (1b)	0.266 [0.122]	0.332 [0.203]	0.080		*90 s (1b)*	0.001 [0.001]	0.001 [0.001]	0.669

	90 s (0b2)	0.279 [0.094]	0.319 [0.170]	0.090		*90 s (0b2)*	0.001 [0.001]	0.001 [0.001]	0.798
	90 s (2b) *	0.262 [0.088]	0.337 [0.198]	0.015		*90 s (2b)*	0.001 [0.002]	0.001 [0.002]	0.576

$^{W}\overline{D}(t)$ – weighted normalized node degree

Transition probability analysis between characteristic brain network states

To characterize the temporal evolution of brain networks, we also adopted the method described in Refs.[1-2] with a priori defined dynamic functional connectivity states, which is fundamentally different from our graph theoretical analysis based dynamic approach. The temporal evolution of brain networks can be captured in a sequence of characteristically different brain states accompanying the simultaneously performed cognitive task. This process is modeled as a Markov-chain as transition into a different brain states only depends on the actual state and occurs with a certain, fix probability. To this end, we investigated age-related differences in transitional probabilities, number of transitions between each dynamic functional connectivity state and dwelling time within each dynamic functional connectivity state.

Here we used temporal evolution of local node degree values characterizing dynamic functional connectivity in 48 brain regions. To assess brain states independent from subject-to-subject variation, $^{W}D_{loc}(t)$ were converted to 48 z-score values for each t (indicating temporal index of analytical window), subject and *n*-back state. For each age group and n-back task difficulty level, we defined a characteristic dynamic functional connectivity state (0-back state→0BS, 1-back state→1BS, 2-back state→1BS) by averaging z-scored $^{W}D_{loc}(t)$ values. Please note that this is different from the method applied in [1-2], where authors identified clusters of brain networks characterized by similar topology using the k-nearest neighbor algorithm. Then we calculated the Euclidean distance between subject- and time-specific standardized local node degree values and the age- and task difficulty-specific averages corresponding to actual and characteristic dynamic functional connectivity states, respectively. Depending on which brain which characteristic state yielded the minimum Euclidean distance, we assigned 0BS, 1BS or 2BS to each subject and actual brain network (varying by t). From the obtained subject-specific sequence of 0BS, 1BS and 2BS, we computed the:

i) transitional probability matrix (probabilities of transition from 0BS to 0BS, 0BS to 1BS, 0BS to 2BS, 1BS to 0BS, 1BS to 1BS, 1BS to 2BS, 2BS to 0BS, 2BS to 1BS, 2BS to 2BS states, respectively),

ii) average dwelling times (average time spent in 0BS, 1BS and 2BS states without transition to another state) and

iii) number of transitions.

Results are reported in **Table S5**. We used Mann-Whitney test for all comparisons due to non-normal distribution of data. We found that translational probabilities are significantly higher in the elderly group compared to the young group for 0BS→0BS, 0BS→1BS, 1BS→0BS in case of all *n*-back session (with the exception of 1BS→0BS during 1-back, with $p>0.05$). In contrast, these transitions are more probable ($p<0.05$) in the younger group: 0BS→2BS, 2BS→0BS, 1BS→1BS, 1BS→2BS, 2BS→1BS, 2BS→2BS. There were no significant differences in dwelling times during any *n*-back sessions. Finally, the number of 0BS→1BS transitions were significantly higher and all other type of transitions were significantly lower in the elderly group compared to the young group for all *n*-back sessions. These differences imply that aged participant's dynamic functional connectivity state is more

associated with to the 0BS reference brain state corresponding to 0-back session, but less associated with the 2BS reference brain state corresponding to 2-back session.

Table S5. *Transition probability analysis of brain states.* Trajectories of local brain network metrics define clusters with characteristically different functional connectivity and was calculated as described in the Supplementary text.

**Transition probabilities**		**Young (Mean±SD)** *→0BS*	*→1BS*	*→2BS*	**Elderly (Mean±SD)** *→0BS*	*→1BS*	*→2BS*
**0-back**	*0BS→*	0.461±0.256	0.003±0.004	**0.005±0.006**	**0.897±0.088**	**0.014±0.010**	0±0
	1BS→	0.033±0.053	0±0	**0.001±0.001**	**0.089±0.079**	0±0	0±0
	2BS→	**0.212±0.118**	**0.067±0.086**	**0.218±0.110**	0±0	0±0	0±0
**1-back**	*0BS→*	0.484±0.286	0.006±0.008	**0.005±0.006**	**0.856±0.129**	**0.018±0.013**	0±0
	1BS→	0.079±0.138	0±0	**0.001±0.001**	0.127±0.118	0±0	0±0
	2BS→	**0.173±0.134**	**0.065±0.098**	**0.188±0.142**	0±0	0±0	0±0
**2-back**	*0BS→*	0.461±0.275	0.003±0.003	**0.008±0.009**	**0.872±0.129**	**0.017±0.014**	0±0
	1BS→	0.032±0.046	0±0	**0.001±0.002**	**0.111±0.120**	0±0	0±0
	2BS→	**0.198±0.106**	**0.100±0.107**	**0.196±0.106**	0±0	0±0	0±0
**Dwelling times**		**Young (Mean±SD)** *0BS*	*1BS*	*2BS*	**Elderly (Mean±SD)** *0BS*	*1BS*	*2BS*
**0-back**		42.43±75.08	1.740±1.254	1.706±0.955	45.92±67.53	1.225±0.627	0±0
**1-back**		22.94±36.65	2.006±1.398	1.612±0.950	30.04±38.67	1.578±0.892	0±0
**2-back**		23.17±37.34	1.468±1.125	2.839±3.607	32.08±39.16	1.240±0.662	0±0
**Number of transitions**		**Young (Mean±SD)** *→0BS*	*→1BS*	*→2BS*	**Elderly (Mean±SD)** *→0BS*	*→1BS*	*→2BS*
**0-back**	*0BS→*	-	6.524±6.933	**9.333±9.962**	-	**13.47±9.705**	0±0
	1BS→	**6.048±6.531**	-	**1.429±2.135**	0.089±0.079	-	0±0
	2BS→	**9.762±10.173**	**1.095±1.786**	-	0±0	0±0	-
**1-back**	*0BS→*	-	4.762±4.784	**4.667±5.170**	-	**8.333±6.250**	0±0
	1BS→	**4.905±4.918**	-	**0.619±1.244**	0.127±0.118	-	0±0
	2BS→	**4.524±5.154**	**0.714±1.384**	-	0±0	0±0	-
**2-back**	*0BS→*	-	2.571±2.315	**7.143±6.995**	-	**8.067±6.464**	0±0
	1BS→	**2.381±2.061**	-	**1.143±1.590**	0.111±0.120	-	0±0
	2BS→	**7.238±6.730**	**0.952±1.431**	-	0±0	0±0	-

0BS – a brain state corresponding to the average functional connectivity during 0-back sessions
1BS – a brain state corresponding to the average functional connectivity during 1-back session
2BS – a brain state corresponding to the average functional connectivity during 2-back session
Bold: significantly higher compared to the corresponding age-comparison group, $p<0.05$

Supplementary references

[1] T. Csipo, A. Lipecz, G. A. Fulop, R. A. Hand, B. N. Ngo, M. Dzialendzik, S. Tarantini, P. Balasubramanian, T. Kiss, V. Yabluchanska, F. Silva-Palacios, D. L. Courtney, T. W. Dasari, F. Sorond, W. E. Sonntag, A. Csiszar, Z. Ungvari, A. Yabluchanskiy, *Geroscience* **2019**, *41* (2), 125

[2] a) K. Wild, D. Howieson, F. Webbe, A. Seelye, J. Kaye, *Alzheimers Dement* **2008**, *4* (6), 428; b) P. Rabbitt, C. Lowe, *Psychol. Res.* **2000**, *63* (3-4), 308
